# CircRNA Itm2b induces oxidative stress via the interaction with Sirt1-Nox4 to aggravate sleep disturbances after traumatic brain injury

**DOI:** 10.1186/s13578-025-01353-6

**Published:** 2025-02-17

**Authors:** Jiayuanyuan Fu, Mengran Du, Biying Wu, Chenrui Wu, Xin Li, Weilin Tan, Xuekang Huang, Ziyu Zhu, Jie Zhang, Zheng Bu Liao

**Affiliations:** 1 Department of Neurosurgery, The First Affiliated Hospital of Chongqing Medical University, No. 1 Youyi Road, Yuanjiagang, Yuzhong District, Chongqing, 400016 China; 2 Department of Neurosurgery, The Second Affiliated Hospital of Chongqing Medical University, Yuzhong District, Chongqing, 400010 China

**Keywords:** CircRNAs, Sleep disorders, Mitochondrial dysfunction, Oxidative stress, Traumatic brain injury

## Abstract

Sleep disorders (SD) are common sequelae following traumatic brain injury (TBI) and may be linked to mitochondrial oxidative stress dysregulation after TBI. Increasing evidence showed that circRNAs play crucial roles in nervous system diseases. However, the involvement of circRNAs in sleep disturbances after TBI is not characterized. In this study, differentially expressed circRNAs were identified by RNA sequencing. Sleep quality in TBI patients was assessed through sleep scales and electroencephalograms. Further experiments were conducted to investigate the role of circItm2b. We found that circItm2b was elevated and involved sleep disorder in TBI patients. Over-expression of circItm2b might aggravate sleep disturbances in mice after TBI. Mechanically, circItm2b regulates Nox4 expression through binding Sirt1, which influences mitochondrial oxidative stress-caused circadian protein losses. Moreover, the knockdown of circItm2b attenuated mitochondrial oxidative stress-induced circadian proteins losses via circItm2b/Sirt1/Nox4 axis after TBI, which might suggest that circItm2b may serve as a prognostic marker for improving sleep disorders and represent a promising therapeutic target for TBI-related sleep disturbances.

**Supplementary Information:**

The online version contains supplementary material available at 10.1186/s13578-025-01353-6.

## Introduction

Traumatic brain injury (TBI) can impair normal brain function and potentially lead to chronic sleep disorders [1]. Sleep disorders following TBI are very common and are extremely prevalent 30 to 84% [2]. The incidence of insomnia following TBI is reported to be nearly 29% [3], while circadian rhythm sleep–wake disorders affect 36% of patients with post-TBI insomnia [4]. Moreover, sleep disturbances increase the risk of cognitive and psychiatric disorders, including cognitive impairment, posttraumatic stress disorder (PTSD), and anxiety following TBI [5]. Despite widespread recognition of sleep disturbance after TBI, the molecular mechanism underlying sleep disorders following TBI has not been elucidated clearly.

Oxidative stress and mitochondrial dysfunction are two critical processes in the pathophysiology of secondary injury following TBI. Some studies and animal models implicated vital insights into the relationship between sleep disorders and mitochondrial oxidative stress [6, 7], such as chronic sleep disorders in rats decreased superoxide dismutase (SOD) activity in the hippocampus [8]. Other studies have highlighted the complicated interdependence between mitochondrial oxidative stress and sleep circadian rhythms, suggesting that alternations in redox status and cellular energetics could influence core clock function [9, 10]. Oxidative damage induced by TBI can result in structural and functional disorders of mitochondria, leading to neuron death and cognition impairment after TBI [11]. Our study aims to investigate the link between mitochondrial oxidative stress and sleep disorders in TBI.

Circular RNAs (circRNAs) are newly recognized types of non-coding RNAs characterized by stable structures and conserved sequences, which may have the potential to interact with RNAs or proteins. Recent studies support that critical role of circRNAs in TBI, with reports of significant differential expression of circRNAs following TBI [12]. Our previous research indicated that circLphn3 could protect the blood–brain barrier damage caused by TBI [13], while melatonin could reduce ferroptosis after TBI through circPtpn14/miR-351-5p/5-LOX axis [14]. Yet, limited studies have explored the relationship between non-coding RNAs and sleep disorders. Several studies have illustrated that miRNAs might be linked to circadian rhythm [15–17]. Si et al. provide the profiles and functions of long non-coding RNAs (lncRNAs) in circadian disorders in Alzheimer’s disease [18]. CircRNA Cdr1as was recently identified as a potential biomarker involved in light–dark cycle entrainment in the suprachiasmatic nucleus (SCN) [19]. However, scarcely any literature reported the association between circRNAs and sleep disorders in TBI. Thus, a notable research gap exists in understanding whether circRNAs are linked to sleep disorders following TBI.

To address this gap, sleep disturbance of TBI patients were assessed by sleep scales and electroencephalograms, and we elucidated that the overexpression of circItm2b was positively associated with sleep disorders in TBI patients. Subsequently, the functions of circItm2b were investigated in mice and HT22 cells. Finally, the circItm2b/Sirt1/Nox4 signaling axis was validated for its involvement in sleep disorders following TBI.

## Methods

### Ethics statement and patient recruitment

The Ethics Committee of the First Affiliated Hospital of Chongqing Medical University approved this study (No. 2019-215), and the research performed in line with the Declaration of Helsinki. Patients or their legally authorized representatives provided written informed consent. All animal experiments were approved by the Chongqing Medical University Animal Care and Use Committee and conducted in accordance with the NIH Guide for the Care and Use of Laboratory Animals published (NO. 2021-177).

TBI patients were recruited from the Neurosurgery department of the First Affiliated Hospital of Chongqing Medical University from September 2019 to December 2021. Totally, fifty patients with acute TBI and 20 healthy people as control were recruited. The inclusion criteria of TBI patients are as follows: (1) aged 18–65 years old; (2) recent head trauma; (3) a pre-admission Glasgow Coma Scale score (GCS) of 8–15; and 4) written informed consent. The exclusion criteria were: (1) medical history of head trauma, cerebral surgery, or other craniocerebral diseases; (2) history of psychiatric conditions; (3) current pregnancy; (4) serious complications such as muti-organ failure; (5) hospitalization for > 1 month; and (6) withdrawal from the study. All clinical data were shown in Supplementary Table 1.

### Preparation of brain specimens and blood samples from patients

Contused brain samples were acquired from six patients who underwent brain contusion operation 5–20 h after brain trauma and control brain samples from three patients undergoing surgery for intraventricular benign tumors (WHO I). During surgery, an approximately 1 cm^3^ sample was obtained. The contusion brain tissue samples were instantly frozen in liquid nitrogen and stored at − 80 °C until the double-labeled immunofluorescence and Western blotting analyses.

In addition, venous blood samples were obtained from 50 TBI patients at admission and from 20 healthy controls. Blood samples were stored at − 80 °C until analysis. The patients involved in this study were not blinded to the treatment.

### Diagnosis of sleep disorders in TBI patients

The diagnosis of sleep disorders after TBI was made by gathering the following data: (1) self-reported sleep disturbance symptoms; (2) results of the subsequent sleep scales one month after TBI; (3) electroencephalography (EEG) recording at 30d after TBI. Pittsburgh sleep quality index (PSQI) and Athens Insomnia Scale (AIS) were used to assess the situation of sleep. The PSQI is a self-report questionnaire with 7 components; a total score greater than 5 indicates poor sleep quality. [20]. The AIS is an 8-item self-report questionnaire used to assess subjective feelings about sleep; an AIS score > 6 indicates insomnia [21]. Patients have performed the Ambulatory electroencephalogram at 30d to assess the sleep architecture. Electrodes were positioned via manufacturers’ instructions and the International 10–20 system for EEG electrode placement for use.

### Animal experiments

Male C57BL/6 mice (weighing 22–25 g, aged 8–10 weeks) were purchased from the Animal Experiment Center of Chongqing Medical University. All animals (total of 240 male mice) were kept at 24 °C and 50 ± 1% humidity under a 12:12 h light/dark cycle with ad libitum access to food and water.

The necessary sample sizes for animal studies were determined employing a sample size calculator (http://www.lasec.cuhk.edu.hk/sample-size-calculation.html). Mice were randomly divided into the following 6 groups: (1) Sham, (2) TBI, (3) TBI + oe-circ-NC, (4) TBI + oe-circItm2b, (5) TBI + sh-circ-NC, and (6) TBI + sh-circItm2b. Fifteen mice in every group were performed the behavioral experiments, and the rest of the mice were performed the following experiments. The experimenters were blind to the animal groupings.

### Injection of lentivirus into the left parietal lobe in mice

The lentivirus to overexpress the circItm2b (oe-circItm2b) and their control (oe-circ-NC) were purchased from GeneSeed (Guangzhou, China). The lentivirus which is to knock down the circItm2b (sh-circItm2b) and their control (sh-circ-NC) were purchased from Hanbio (Shanghai, China).

To anesthetize the mice with isoflurane (5%) and secured them in the stereotaxic frame. Subsequently, to perform three injections into the left parietal cortex, the coordinates were as following: AP 1.0 mm, L 1.5 mm, H 1.0 mm; AP 1.0 mm, L 2.5 mm, H 1.0 mm; AP 1.5 mm, L 2.0 mm, and H 1.0 mm (David Kopf Instruments, Tujunga, CA, USA). Three locations in the left parietal cortex were injected using lentivirus in PBS (3 μL), which also served as the controlled cortical impact (CCI) model injury area 14 days later.

### Controlled cortical impact (CCI) mice model

Establishment of CCI mice has been previously described [14]. Briefly, for the TBI group, mice were anesthetized with 5% isoflurane and sustained anesthesia in oxygen-enriched air (20% oxygen/80% air). The head of each mouse was secured in a stereotaxic frame and impact occurred with a velocity of 5.0 m/s, a depth of 1.5 mm, and a 100 ms dwell time. For the sham condition without cortical impact injury, only craniotomy was performed to remove the skull.

Successful CCI model mice were chosen for the subsequent experiments based on the results of the grasping test of the contralateral limb 2 h post-TBI. The grasping test method was performed as before [22]. Briefly, let mice grasp the bars of the cages, and when the signs of finger flexion are noticed once, then assess for grasping strength. The successful model was judged by the grasping strength, which means the low grasping strength on the bar of cages represented success.

### Modified neurological severity score test (mNSS)

The modified neurological severity score test was performed as our previous study [23]. The test included motor, sensory, balance, reflex, and abnormal movement parts. The score between 1 and 6 points represents mild damage, the score between 7 and 12 points represents moderate damage, and the score between 13 and 18 points represents severe damage.

### Electroencephalogram (EEG) recording in TBI mice

Chloral hydrate (3.5%) was used to induce sleep via intraperitoneal injection (Macklin, Cat#C804539, China, 5 ml/kg) before EEG monitoring (Natus Medical, USA). To examine post-TBI sleep architecture, EEG was recorded on days 14 and 30 after TBI. After implanting the system ground electrode, two active electrodes and two reference electrodes were implanted respectively on the ipsilesional and contralesional parietal cortex and on the mastoid process. The parameters are described in detail in our previous study [14]. EEG viewer (Natus Medical, USA) was used to analyze the wave alternations (α, β, θ, and δ waves) between groups.

### Cell cultures and transient transfection

Mouse Hippocampal neuronal cells (HT22 cells) and 293 T cells were purchased from OTWO BIOTECH (Shenzhen, China). To culture the HT22 cells and the 293 T cells using low glucose DMEM 10% fetal bovine serum and high glucose DMEM 10% fetal bovine serum respectively, at 37 °C in a humidified atmosphere containing 5% CO_2_. The overexpression and the knockdown of Sirt1 were purchased from Tsingke Biotechnology Co. (Beijing, China). Lipofectamine™ 2000 (Thermo Fisher Scientific, USA) was used in accordance with the protocol of manufacturers to transfect the lentivirus or the plasmids. QRT-PCR was performed after 7d of lentivirus transfection to examine the efficiency. We use 600 μM/L as the concentration of H_2_O_2_ in the culture medium, treating with the H_2_O_2_ for 6 h.

### Isolating mitochondria

To isolate mitochondria from HT22 cells or brain specimens, they were executed in accordance with the manufacturer’s protocol (Nanjing Jiancheng, Nanjing, China). Measurement of mitochondrial ATP was performed according to the manufacturer’s recommended protocol (Nanjing Jiancheng, Nanjing, China). The method of luciferin chemiluminescence was employed for the measurement. Cells or tissues were homogenized in lysis buffer. Centrifuging the lysates at 4 °C at 12,000 × g for 10 min. To collect the supernatants for the following test. The protein assay kit (Nanjing Jiancheng, Nanjing, China) was used to quantify the concentrations and convert the concentration of ATP into nmol/mg.

### Intracellular ROS assay

The MitoSOX™ Red Mitochondrial Superoxide Indicator Kit was purchased from Yeasen (Shanghai, China) and used to detect ROS levels of mitochondria in HT22 cells. First, a 5 μM working stock solution of MitoSOX™ Red reagent was added to the HT22 cells. The cells were then incubated in the dark at 37 °C for 10 min, washed, and resuspended in 500 μl of PBS. The laser-scanning confocal microscope (Zeiss LSM800, Germany) was used to assess ROS levels.

The mitochondrial ROS level (Nanjing Jiancheng, Nanjing, China) was measured in line with the manufacturer’s recommended protocol. Briefly, after treatment, the brain tissue or cells were washed with PBS, and added the DCFH-DA working solution, then incubated at 37 °C for 30 min. Wash 1–2 times using PBS followed by measuring intensity.

### RNA extraction, RNA-seq and bioinformatics analysis

The RNA extraction and RNA-seq procedures were described in detail in our previous study [23]. Briefly, after the extraction of total RNA, RNA sequencing was performed using the BGI platform (BGI-Shenzhen, China). The sequencing data were filtered with SOAPnuke (v1.5.2) [24]. The clean reads were then mapped using HISAT2 (v2.0.4) [25]. The Bowtie2 (v2.2.5) [26] was used to align the clean reads to the reference coding gene set, and the expression level of the gene was calculated by RSEM [27] (v1.2.12). The heatmap was drawn using the “pheatmap” package in R. Differential expression analysis was performed by DESeq2 (v1.4.5) [28] with Q value ≤ 0.05 and the |Fold Change|> 1 as the threshold of differential expression. The circBase (http://www.circbase.org/) was used to obtain the sequences of circRNA and to analyze the homology of circRNA between humans and mice.

### Transmission electron microscopy

The ipsilesional cortex tissues (1 mm^3^) were prepared according to the previous study [29]. In brief, the brain samples were fixed for 48 h in glutaraldehyde (4%), and to immerse the brain tissues for 1 h in 1% osmium tetroxide, then dehydrated in ethanol. To collect the samples on the grids after the brain samples were cut into 70-nm sections. Using the lead citrate and uranyl acetate to stain the grids, then using the transmission electron microscope (JEOL JEM-1400PLUS, Japan) to observe.

### Quantitative real-time PCR (qRT-PCR)

The total RNA of brain tissues and HT22 cells was extracted using the RNA Extraction Kit (Qiagen, RNeasy mini Kit) and then mixed with the reverse transcription reagent (RT Master Mix for qPCR Kit, MedChemExpress, USA); all steps were performed according to the manufacturer’s protocol. Treatment of RNase R (Beyotime, China) was performed for 15 min at 37 °C. RT Master Mix of the qPCR Kit (MCE, USA) was used to reverse transcribe the RNA into cDNA. SYBR Green qPCR Master Mix (MCE) was used for qRT-PCR. The primer sequences are shown in Table 1.

**Table 1:** The primers for qRT-PCR

Target	Sequence (5′ → 3′)
circItm2b (divergent)	F: ACTGTTTCACCATTCGGCAT R: ACGTCACCCGGGTCATGAA
circItm2b (convergent)	F: TACCAGAGTTTGCGGACAGC R: AGTTACTGGCTTCCCGCTTC
*Itm2b*	F: TGTGCCTGTACCAGAGTTTGC R: CTTCCCGCTTCTGAATACCTC
hsa_circ_0006620	F: GGTGTTGGTGCATGTGCTTT R: AAACTCTGGGACAGGCACAC
mmu_circ_0011676	F: GCATCCCAACTCAATGTCAGC R: TTCGTAGGGTGGGTAGCTCA
mmu_circ_0008937	F: ACAACCTTAAGCAGACAGTGATG R: TTGACCTTCTCCCGGAACAC
mmu_circ_0009294	F: AGAGACCCAATGTCTGTGGA R: ATCCATCCCCACAGGAATGC
mmu_circ_0003725	F: AAGTCTGAGGCCATTGGTTCTA R: AACACTGTCGTGAGTTGGTGA
*NOX4* (human)	F: AGATGTTGGGGCTAGGATTG R: TCTCCTGCTTGGAACCTTCT
*CLOCK* (human)	F: ACGAGAACTTGGCATTG R: GTTGGTGTTGAGGAAGG
*BMAL1*(human)	F: TTAGCCAACGTCCTGGAAGG R: CCTTCTCCAGAGGGCAGCAT
gapdh (mouse)	F: AGGTCGGTGTGAACGGATTTG R: TGTAGACCATGTAGTTGAGGTCA
*GAPDH* (human)	F: GGAGCGAGATCCCTCCAAAAT R: GGCTGTTGTCATACTTCTCATGG

### Immunofluorescence and fluorescence in situ hybridisation

First, the brain slices (ipsilesional cortex) and HT22 cells were rinsed in TBST and pretreated in antigen unmasking solution for 1 h. The slices and cells were then incubated with the primary antibodies at 4 °C overnight. The next day, they were incubated with the secondary antibody for 1 h at 37 °C and then rinsed in TBST twice for 5 min. The antibodies used were as follows: Sirt1 (Zen bio, cat#340929), Nox4 (Proteintech, cat# 67681-1-Ig), Clock (GeneTex, cat#GTX102318), and Bmal1 (GeneTex, cat#GTX105060). The fluorescence in situ hybridization (FISH) was acted in accordance with the protocol of the kit (RiboBio). The probes of circItm2b in the FISH experiment were purchased from GeneSeed (China). Briefly, cells were fixed using paraformaldehyde and added to the prehybridization solution, then incubated for 30 min at 37 °C. The hybridization solution which contains the FISH probes were incubated at 37 °C overnight in the dark. Images were obtained using a fluorescence microscope (Zeiss LSM800, Germany).

### Western blotting analysis

Proteins extracted from brain tissues (ipsilesional cortex) or cells were separated by SDS-PAGE and transferred to a PVDF membrane. The PVDF membrane was incubated with primary antibodies (1:1000) at 4 °C overnight. Then the blots were incubated with the secondary antibodies (1:8000) for 1 h. ImageJ software was used to quantify relative expression. The antibodies used were as follows: Nox4 antibody (Zen bio, cat#R380874), Cytc C (Zen bio, cat#250109) antibody and Cox IV (Zen bio, cat#200147) antibody, and β-Actin (GeneTex, cat#GTX109639, USA) were the internal references. Other antibodies have been mentioned before.

### Pull-down assay

Pull-down assay was carried out as we previously described [30]. Biotinylated circItm2b back splice probes and mutant probes (mut1, mut2, and mut3) were designed and synthesized by GeneSeed (Guangzhou, China). Briefly, the capture buffer was used to treat cells and to gain the supernatant, then the magnetic beads (MCE, USA) was used to incubate with the probes with rotation at 4 °C. To wash the collected beads twice using the capture buffer. The bead coated with probes were mixed with the supernatant with rotation at 4 °C for 1 h. Then, to wash the beads for three times. The RNA–protein complexes bound to beads were isolated for the next western blotting analysis.

### RNA immunoprecipitation (RIP) assay

RIP kit (Millipore, USA) was used to perform the RIP assay which verified the interaction between circItm2b and Sirt1. In brief, collecting and lysing the HT22 cells using the RIP lysis buffer. The RNA interaction was precipitated using the antibody of anti-Sirt1 (ab189494, Abcam), and for the negative control, the anti-Ig-G antibody (ab172730, Abcam) was employed. Then analyzed by qRT-PCR.

### Luciferase assay

The sequence of Nox4 promotor was constructed into the psiCHECK2 plasmid (HanBio) and created the reporter constructs with putative Sirt1 binding sites. Subsequently, 293T cells were transfected with reporter vectors, and the Sirt1 plasmid. The Dual-Lumi™ II Luciferase Reporter Gene Assay Kit (BeyoTime, China) was used to measure the Firefly and Renilla luciferase activities. The result was normalized to the ratio between Renilla luciferase activity and firefly luciferase activity.

### Statistical analysis

The data was conducted the statistical analysis by GraphPad Prism 9 (Version 9.1.1). The normality test was performed. Two independent sets of samples were compared using the two-tailed t-test. In cases of more than two groups, one-way ANOVA or two-way ANOVA analysis followed by Tukey’s post hoc test was used for comparisons. Results are shown as the mean ± standard deviation (SD). The level of statistical significance was set as p < 0.05.

## Results

### The profile of circItm2b was identified in mice post-TBI and H_2_O_2-_treated cells

The circRNA expression profiling after TBI was revealed after RNA-seq. All sequencing data were uploaded to NCBI (PRJNA725662). Secondary brain injury is a major focus in neurosurgery, as it is crucial for the treatment of brain injuries. Studies have reported the pathophysiological alternations peak on day 3 after TBI [31]. Therefore, we selected the 3d after TBI at the time point for sequencing.

**Fig. 1:**
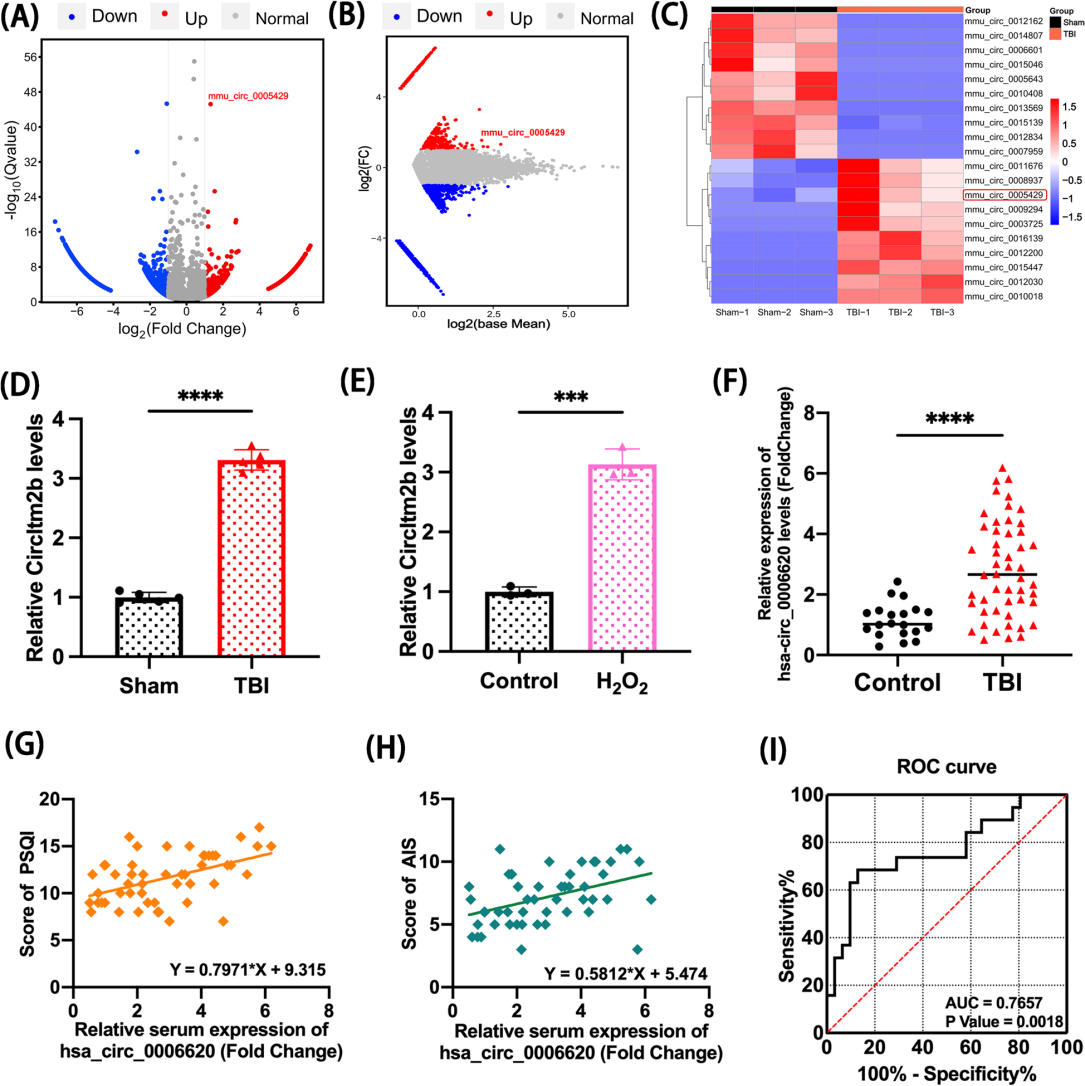
Elevated circItm2b expression in mice TBI and H_2_O_2_-treated HT22 cells, and hsa_circ_0006620 that increased in TBI patients might involve in sleep disorders after TBI. **A** The volcano plot of differentially expressed circRNAs between sham and TBI groups, the timepoint of RNA-seq is 3d after TBI. (Fold Change = 1, Q < 0.05). **B** The MA plot shows the differential expression of circRNAs between sham and TBI groups, the timepoint of RNA-seq is 3d after TBI.** C** Clustering heatmap of differentially expressed circRNAs screening by DESeq2, showing the 20 circRNAs with maximum and minimum Q valve, the red rectangle shows the position of mmu_circ_0005429. **D** The relative expression of circItm2b was detected by qRT-PCR between sham and TBI mice on day 3 post-TBI. n = 5 mice per group. **** p < 0.0001, two-tailed t-test. **E** The relative expression of circItm2b was detected by qRT-PCR between control and H_2_O_2_-treated HT22 cells. n = 3. *** p < 0.001, two-tailed t-test. **F** The relative expression of hsa_circ_0006620, which was highly homologous to circItm2b, was increased in the serum of TBI patients. n = 50 TBI patients, n = 20 control volunteers, **** p < 0.0001, two-tailed t-test. **G** The sleep quality was assessed by the Pittsburgh sleep quality index (PSQI) scale, the scale score is positively correlated with the expression of hsa_circ_0006620. n = 50 TBI patients, n = 20 control volunteers, p = 0.0004, two-tailed t-test. **H** The sleep quality was assessed by the Athens Insomnia Scale (AIS), the scale score is positively correlated with the expression of hsa_circ_0006620, n = 50 TBI patients, n = 20 control volunteers, p = 0.0018, two-tailed t-test. **I** ROC curve to diagnose sleep disorders after TBI based on the expression of serum hsa_circ_0006620. All data were represented as mean ± SD

Of the 16,127 circRNAs detected (Fig. 1A, B), 475 were differentially expressed after TBI. Mmu_circ_0005429 (circItm2b) had the lowest Q value, indicating it had the strongest statistical significance among the top five differentially expressed circRNAs (Fig. 1C). We subsequently measured the expression levels of the top five upregulated circRNAs in vivo and in vitro. The results showed that mmu_circ_0005429 was significantly up-regulated after TBI in mouse brain and H_2_O_2_-treated HT22 cells ( Fig. 1D, E). In contrast, the other four differentially expressed circRNAs did not show as significant expression changes as mmu_circ_0005429. The detailed expression patterns of these other circRNAs can be seen in Supplementary Fig. 1A, B. These findings further validate the pivotal role of mmu_circ_0005429 in TBI.

### Elevated hsa_circ_0006620 expression in the serum of TBI patients as a potential biomarker for sleep disorders related to rhythmic protein expression and dynamic EEG changes

A few studies have reported that circRNAs are associated with sleep-related diseases [32], yet, few studies directly demonstrate the role of circRNAs in sleep disorders post-TBI. Our results showed that *Itm2b* and *ITM2B* were the parental genes of mmu_circ_0005429 and the hsa_circ_0006620 respectively. In the homology analysis, the identity reached 89.2% between mmu_circ_0005429 and hsa_circ_0006620, according to the BLAT tool in circBase (Table 2). The expression level of hsa_circ_0006620 was up-regulated in the TBI patient’s serum (p < 0.0001, Fig. 1F). Furthermore, hsa_circ_0006620 was positively correlated with PSQI and AIS scores (Fig. 1G, H). Additionally, the receiver operating characteristic (ROC) curve showed that the Area Under Curve (AUC) was 0.7657 (Fig. 1I).

**Table 2:** Homology identity of circItm2b between mice and human

Query	STRAT	END	IDENTITY	circRNA	STRAND	START	END
mmu_circ_0005429	151	128	89.20%	hsa_circ_0006620	+	6125	6317
mmu_circ_0005429	146	461	87.70%	hsa_circ_0006620	+	1246	3754
mmu_circ_0005429	26	692	93.60%	hsa_circ_0027605	−	2729	2769
mmu_circ_0005429	26	692	93.60%	hsa_circ_0027608	−	764	804
mmu_circ_0005429	22	691	95.90%	hsa_circ_0012781	+	136,709	136,741
mmu_circ_0005429	21	667	65.30%	hsa_circ_0035325	−	67,168	67,191

**Fig. 2:**
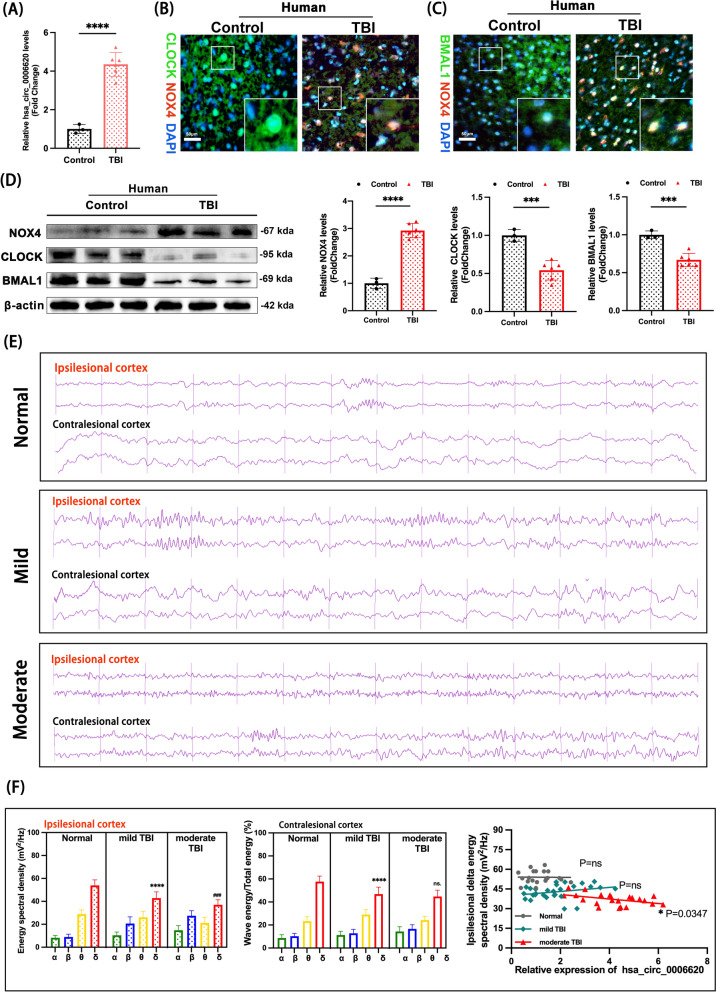
Increased Nox4 level, losses of rhythm proteins, and sleep architecture disturbance in TBI patients.** A** Relative expression of hsa_circ_0006620 in the brain specimens from acute TBI patients, TBI patients n = 6, controls n = 3. TBI vs. control, **** p < 0.0001. **B, C** Representative images of double immunofluorescence show the co-localization of NOX4, CLOCK, and BMAL1 in patients with acute TBI. **D** The expressions of NOX4, CLOCK, and BMAL1 are detected by western blotting in TBI patients. TBI patients n = 6, controls n = 3. NOX4: TBI vs. Control group, **** p < 0.0001; CLOCK: TBI vs. Control group, *** p < 0.001; BMAL1: TBI vs. Control group, *** p < 0.001. One-way ANOVA followed by Tukey’s multiple comparisons test. **E** Representative EEG waves of normal, mild TBI, and moderate TBI groups. Mild TBI group, n = 28; moderate TBI group, n = 22; normal group, n = 20. TBI. Patients underwent EEG recordings 30 days after TBI. **Note:** In the normal group, no injury was present on either side, serving as a baseline for comparison. For statistical analysis, the corresponding side of the normal group was used to compare with the injured side of the experimental groups. **F** The nest histogram shows the percentage of each wave energy (alpha, beta, theta, and delta) relative to the total energy in sleep duration in both the ipsilesional and contralesional cortex at 30d in TBI patients in different groups. Mild TBI group, n=28; moderate TBI group, n=22; normal group, n=20. Ipsilesional cortex (left panel): Mild TBI delta vs. Normal group delta, **** p<0.0001; moderate TBI delta vs. Mild TBI delta, ^###^ p<0.001; contralesional cortex (middle): Mild TBI delta vs. Normal group delta,**** p<0.0001; Moderate TBI delta vs. Mild TBI delta, ns not statistically significant, one-way ANOVA followed by Tukey’s multiple comparisons test. The correlation analysis (right panel) between delta energy density in sleep duration and expression of hsa_circ_0006620 in the serum of TBI patients are shown. Grey points and lines represent the normal group, normal group correlation: p=ns (not statistically significant); green points and lines represent the mild TBI group (GCS 13-15), mild TBI group correlation: p=ns (not statistically significant); red points and lines represent the moderate TBI group (GCS 8-12), moderate TBI group correlation: p=0.0347. The delta energy density in sleep duration negatively correlated with the expression level of hsa_circ_0006620 in the moderate TBI group. All data were represented as mean ± SD

The expression of hsa_circ_0006620 in contused brain tissue from TBI patients was elevated (Fig. 2A). Nox4, a member of the NADPH oxidase (NOX) family, produces superoxide like other NOX isoforms and plays a significant role in oxidative stress after TBI [33]. Circadian locomotor output cycles kaput (Clock) and brain and muscle arnt-like 1 (Bmal1) were heterodimers that drive all circadian pacemakers [34]. Next, the changes of *NOX4, CLOCK* and *BMAL1* in mRNA and protein level were examined in the brain specimens of TBI patients. Our findings showed a marked increase in *NOX4* mRNA expression in TBI samples compared to controls (Supplement Fig. 1C). In contrast, *CLOCK* and *BMAL1* mRNA levels were significantly downregulated in TBI samples (Supplement Fig. 1C), which was consistent with the protein level changes observed in the western blot and immunofluorescence assays (Fig. 2B–D, Supplement Fig. 1D, E). These suggests that the alterations in NOX4, CLOCK, and BMAL1 expression may be related to the disruption of circadian rhythms following TBI.

We then separated the TBI patients into mild and moderate TBI groups according to GCS score at admission. Representative EEG recordings were shown in Fig. 2E. Delta waves, where they constitute the primary component of slow waves, are crucial for restorative sleep and brain recovery. They are the hallmark of deep sleep, especially during deeper stages of sleep [35]. These waves are crucial for restorative sleep and brain recovery. In our study, we observed significant changes in δ wave energy, which showed a notable negative correlation with circItm2b expression levels. Specifically, higher levels of circItm2b expression were associated with lower δ wave energy, suggesting that circItm2b may play a role in modulating deep sleep in TBI patients and could potentially contribute to sleep disturbances (Fig. 2F).

In addition, we analyzed the relationship between circItm2b expression and α wave and β wave activities, which are typically associated with different wakefulness states. Alpha waves are linked to calm, relaxed wakefulness. while beta waves are associated with active mental states and heightened focus. Interestingly, no significant correlation was found between circItm2b expression and α wave activity, indicating that circItm2b does not significantly affect relaxed, calm wakefulness states (Supplementary Fig. 1F). However, we observed a notable increase in β wave activity, suggesting that circItm2b expression may be linked to heightened arousal levels (Supplementary Fig. 1G). Increased β wave activity is often associated with higher vigilance and reduced sleep depth, which can make individuals more prone to waking up during sleep.

Taken together, our analysis suggests that circItm2b expression in TBI patients may be associated with disruptions in both deep sleep (as reflected by δ wave activity) and wakefulness-related states (as reflected by β wave activity). The reduction in δ wave energy and the increase in β wave activity suggest that circItm2b may play a role in modulating the sleep–wake cycle, promoting a heightened state of alertness and vigilance while compromising sleep stability. These findings highlight the potential of circItm2b in regulating sleep quality, especially in patients with sleep disturbances, such as those with TBI.

### Over-expression of circItm2b mediated disorders of sleep in TBI mice

**Fig. 3:**
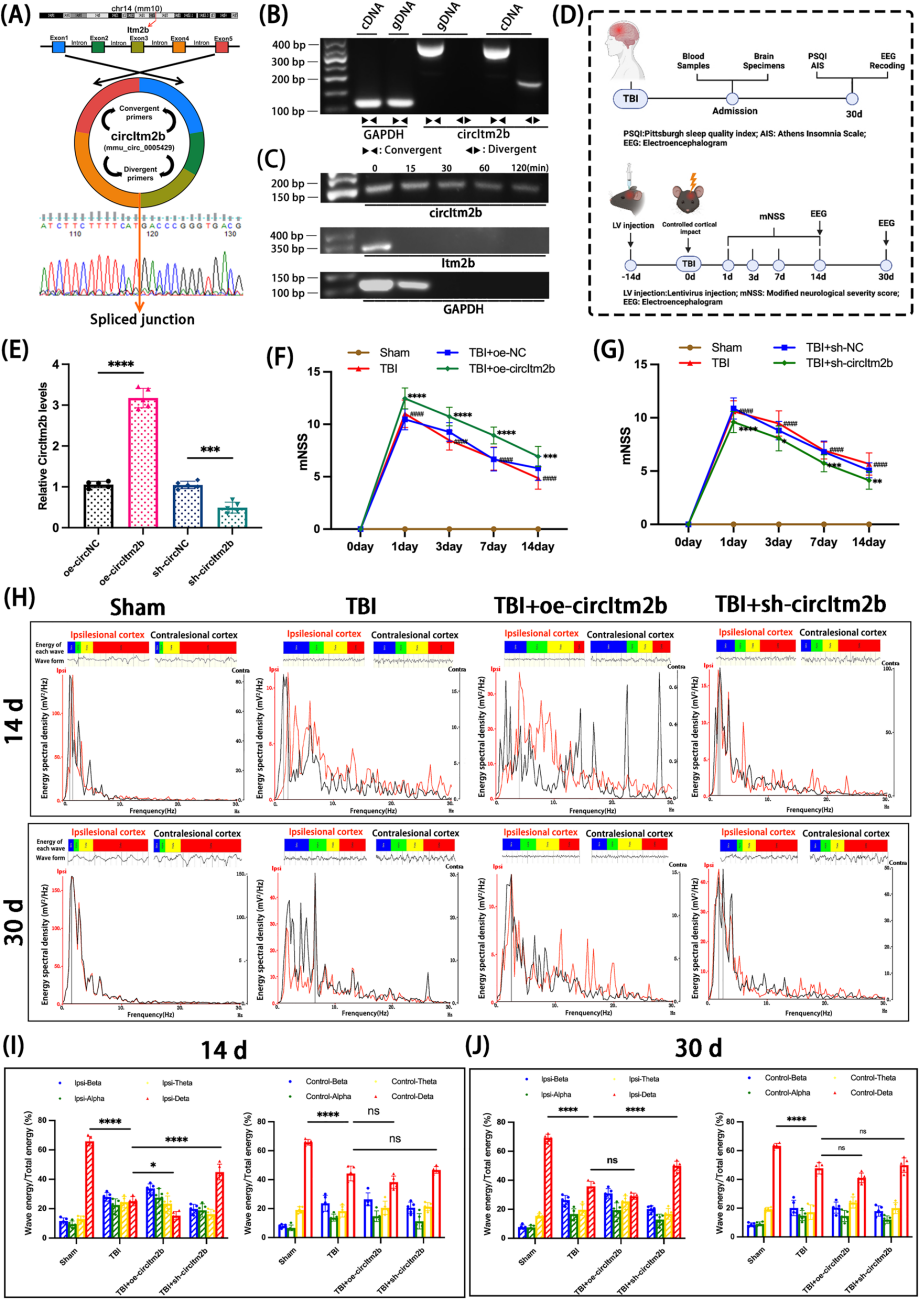
CircItm2b is linked to sleep disorders after TBI in mice. **A** The schematic diagram shows that circItm2b consists of 5 exons. The Sanger sequencing result demonstrates the spliced junction and circularized position. **B** The presence of circItm2b was validated in HT22 cells by RT-PCR and agarose gel electrophoresis (AGE). **C** The circItm2b and linear Itm2b mRNA were treated with RNase R for 0 to 120 min followed by detection through PCR and AGE. **D** Study flow chart of human and animal experiments. **E** The expression efficiency of injection of lentivirus carrying an overexpression or inhibition circItm2b at left parietal lobe in mice 14 days after injection. n = 5 mice per group. oe-circItm2b vs. oe-circNC, **** p < 0.0001; sh-circItm2b vs. sh-circNC *** p < 0.001. **F** Modified Neurological Severity Scores (mNSS) were performed at 0, 1, 3, 7, and 14d after TBI. n = 15 mice per group, TBI vs. the Sham, ^####^ p < 0.0001; TBI + oe-circItm2b vs. TBI + oe-NC, **** p < 0.0001, *** p < 0.001. **G** Modified Neurological Severity Scores (mNSS) were performed at 0, 1, 3, 7, and 14d after TBI. n = 15 mice per group, TBI vs. the Sham, ^####^ p < 0.0001; TBI + sh-circItm2b vs. TBI + sh-NC, **** p < 0.0001, *** p < 0.001, ** p < 0.01, * p < 0.05. **H** Representative energy spectral density and waveforms in the ipsilesional and contralesional cortex were recorded by EEG in different groups at 14d and 30d. Blue: The different color in energy of each wave: blue reperesents beta wave, green reperesents alpha wave, yellow reperesents theta wave and red reperesents delta wave. **Note**: In the sham group, no injury was present on either side, serving as a baseline for comparison. For statistical analysis, the corresponding side of the sham group was used to compare with the injured side of the experimental groups. **I** The histogram shows the percentage of each wave energy (alpha, beta, theta, and delta) relative to the total energy in both the ipsilesional (left panel) and contralesional cortex (right panel) at 14d after TBI. n = 5 mice per group. Ipsilesional: TBI vs. the Sham group, **** p < 0.0001, TBI + oe-circItm2b vs. TBI, * p < 0.05, TBI + sh-circItm2b vs. TBI, **** p < 0.0001. Contralesional: TBI vs. the Sham group, **** p < 0.0001, TBI + oe-circItm2b vs. TBI, ns not statistically significant, TBI + sh-circItm2b vs. TBI, ns not statistically significant; two-way ANOVA followed by Tukey’s test. **J** The histogram shows the percentage of each wave energy (alpha, beta, theta, and delta) relative to the total energy in both the ipsilesional (left panel) and contralesional cortex (right panel) at 30d. n = 5 mice per group. Ipsilesional: TBI vs. the Sham group, **** p < 0.0001, TBI + oe-circItm2b vs. TBI, ns not statistically significant, TBI + sh-circItm2b vs. TBI, **** p < 0.0001. Contralesional: TBI vs. the Sham group, **** p < 0.0001, TBI + oe-circItm2b vs. TBI, ns not statistically significant, TBI + sh-circItm2b vs. TBI, ns not statistically significant; two-way ANOVA followed by Tukey’s test. All data were represented as mean ± SD

The schematic diagram showed the back splicing junction of circItm2b (mmu_circ_0005429), and Sanger sequencing further validated the circularized sites (Fig. 3A). CircItm2b, which has a full length 781 bp and consists of 5 exons of Itm2b in chromosome 14, could be amplified by divergent primers in complementary DNA (cDNA), but not by divergent primers in genomic DNA (gDNA). RNase R treatment could digest linear Itm2b instead of circItm2b (Fig. 3B, C).

The time course of humans or animals in the study was shown in Fig. 3D. Lentivirus injection was performed in the left parietal cortex of mice 14 days before TBI, which is aimed to overexpress or knock down the circItm2b. QRT-PCR confirmed overexpression and knockdown after injection (Fig. 3E). The modified neurological severity score (mNSS) peaked at 1d and declined gradually over time. Notably, the scores in the TBI + oe-circItm2b group significantly increased, however, the scores reduced after knocking down the circItm2b after TBI (Fig. 3F, G). These results indicated that knocking down circItm2b may alleviate neurological injury.

The electroencephalogram (EEG) results of mice were shown in Fig. 3H. On day 14, the delta wave energy spectral densities sharply decreased after TBI both on the ipsilesional and contralesional cortex (Fig. 3I). Whereas, on the ipsilesional cortex, the delta wave showed a worsening reduction when over-expressed the circItm2b and knockdown of circItm2b ameliorated the decreasing of delta wave (Fig. 3I). On the contralesional cortex in 14d EEG results, the delta wave has no statistical significance after overexpressing or knocking down of circItm2b (Fig. 3I). The consequences of the 30d EEG recording showed a similarity to the EEG at 14d, for the delta wave, even though it seems the decreasing delta wave attenuated at 30d compared to day 14, which still had a similar trend of delta reduction after TBI, and deteriorated when overexpressed circItm2b (Fig. 3J), which suggested that the sleep disorders might exist persistently over time.

Additionally, we performed statistical analyses of α waves and β waves to evaluate their relationship with circItm2b expression. The statistical analyses of α waves and β waves in EEG were shown in Supplement Fig. 2. On day 14, the alpha wave energy has no significance on the ipsilesional and contralesional cortex regardless of whether circItm2b was overexpressed or knockdown (Supplement Fig. 2A, B). However, on the ipsilesional cortex, overexpression of circItm2b significantly increased the proportion of β waves, while knockdown of circItm2b significantly decreased the proportion of β waves, the contralesional cortex showed no significance in β waves (Supplement Fig. 2C, D). The outcomes of the 30d EEG recording also indicated a similarity to the EEG at 14d, the alpha waves was also showed no significance on both ipsilesional and contralesional cortex (Supplement Fig. 2E, F), but the beta waves demonstrated the reduction after knockdown the circItm2b (Supplement Fig. 2G, H). These findings indicate that circItm2b does not significantly affect α wave activity, which is typically associated with relaxed, calm wakefulness, and highlight that circItm2b influences β wave activity, which is associated with higher alertness and active mental states, potentially contributing to sleep instability and increased vigilance in TBI conditions.

### Increased circItm2b exacerbated mitochondrial disturbances and oxidative stress-induced rhythm protein losses after TBI

**Fig. 4:**
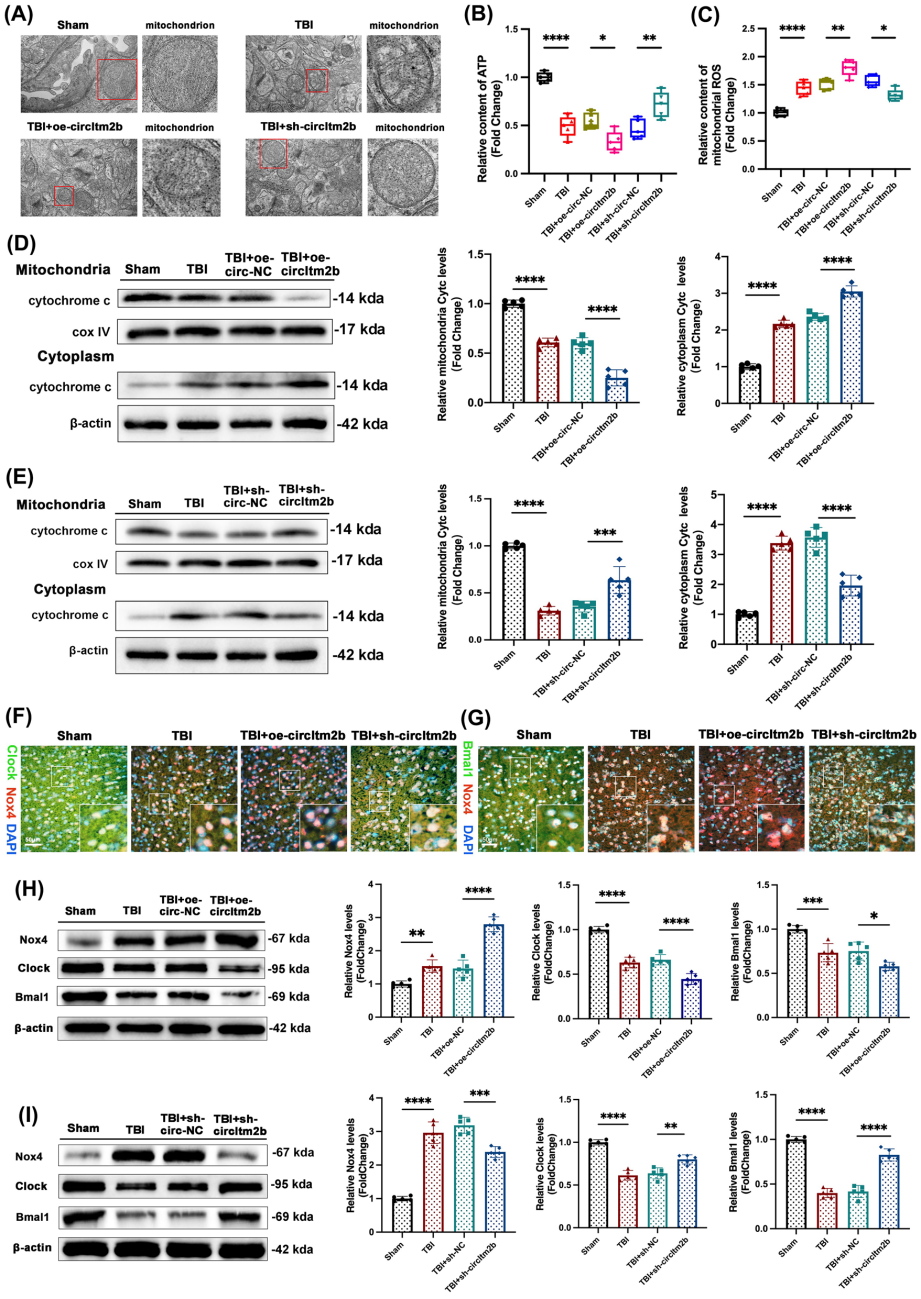
Increased circItm2b exacerbated disturbances of mitochondria and oxidative stress-induced rhythm protein losses. **A** The transmission electron microscope micrograph shows the mitochondrial damages at 3d after TBI, overexpression of circItm2b aggravates the injury, while knockdown of circItm2b alleviates it, and the red rectangle regions indicate the mitochondria. **B** The relative content of ATP after overexpression or inhibition of circItm2b in mice 3 days after TBI. n = 5 mice per group, TBI vs. the Sham group, **** p < 0.0001; TBI + oe-circItm2b vs. TBI + oe-circ-NC, * p < 0.05; TBI + sh-circItm2b vs. TBI + sh-circ-NC, ** p < 0.01. One-way ANOVA followed by Tukey’s multiple comparisons test. **C** The relative content of mitochondrial ROS level after overexpression or inhibition of circItm2b in mice 3 days after TBI. n = 5 mice per group, TBI vs. the Sham group, **** p < 0.0001; TBI + oe-circItm2b vs. TBI + oe-circ-NC, ** p < 0.01; TBI + sh-circItm2b vs. TBI + sh-circ-NC, * p < 0.05. One-way ANOVA followed by Tukey’s multiple comparisons test. **D** Western blot bands of cytochrome C from the mitochondria to the cytoplasm with circItm2b overexpression in the brain of mice on 3 days after TBI, n = 5. Mitochondria: TBI vs. Sham group, **** p < 0.0001; TBI + oe-circItm2b vs. TBI + oe-circ-NC, **** p < 0.0001; cytoplasm: TBI vs. Sham group, **** p < 0.0001, TBI + oe-circItm2b vs. TBI + oe-circ-NC, **** p < 0.0001. **E** Western blot bands of cytochrome C from the mitochondria to the cytoplasm with circItm2b knockdown in the brain of mice on 3 days after TBI, n = 5. Mitochondria: TBI vs. Sham group, **** p < 0.0001; TBI + sh-circItm2b vs. TBI + sh-circ-NC, *** p < 0.001; cytoplasm: TBI vs. Sham group, **** p < 0.0001, TBI + sh-circItm2b vs. TBI + sh-circ-NC, **** p < 0.0001. One-way ANOVA followed by Tukey’s multiple comparisons test. **F, G** The representative images of double immunofluorescence show the co-localization of Nox4, Clock, and Bmal1 in mice after overexpression or knockdown of circItm2b. **H** The expressions of Nox4, Clock and Bmal1 are detected by western blotting after circItm2b overexpression. n = 5 mice per group. Nox4: TBI vs. Sham group, ** p < 0.01, TBI + oe-circItm2b vs. TBI + oe-circ-NC, **** p < 0.0001; Clock: TBI vs. Sham group, **** p < 0.0001, TBI + oe-circItm2b vs. TBI + oe-circ-NC, **** p < 0.0001; Bmal1: TBI vs. Sham group, *** p < 0.001, TBI + oe-circItm2b vs. TBI + oe-circ-NC, * p < 0.05. One-way ANOVA followed by Tukey’s multiple comparisons test. **I** The expressions of Nox4, Clock and Bmal1 are detected by western blotting after circItm2b knockdown. n = 5 mice per group. Nox4: TBI vs. Sham group, **** p < 0.0001, TBI + sh-circItm2b vs. TBI + sh-circ-NC, *** p < 0.001; Clock: TBI vs. Sham group, **** p < 0.0001, TBI + sh-circItm2b vs. TBI + sh-circ-NC, ** p < 0.01; Bmal1: TBI vs. Sham group, **** p < 0.0001, TBI + sh-circItm2b vs. TBI + sh-circ-NC, **** p < 0.0001. One-way ANOVA followed by Tukey’s multiple comparisons test. All data were represented as mean ± SD

Mitochondrial dysfunction and oxidative stress were essential processes in secondary injury after TBI [36]. Multiple pathological processes are involved in the pathophysiology of TBI, and ultrastructural alternations are one feature of brain damage [37]. Therefore, transmission electron microscopy revealed that the mitochondrial membrane was swollen and ruptured post-TBI, which was aggravated by overexpressing circItm2b; however, this process was altered in reverse when circItm2b was knocked down (Fig. 4A). Moreover, overexpression of circItm2b reduced the content of ATP and increased the mitochondrial ROS level compared to the TBI group, suggesting aggravated mitochondrial oxidative stress. In contrast, knocking down circItm2b alleviated this process (Fig. 4B, C). We then detected the expression of cytochrome C by Western blotting. The results showed that in the TBI + oe-circItm2b group, the cytochrome C was reduced and translocated from the mitochondria to the cytoplasm (Fig. 4D), while circItm2b knockdown reversed this effect (Fig. 4E). Hence, inhibiting circItm2b expression could alleviate mitochondrial ultrastructural damage and oxidative stress.

Then, the expression of Nox4, Clock, and Bmal1 were detected to identify how circItm2b exerted its function in TBI and in subsequent sleep disorders after TBI. Our results found that overexpressing the circItm2b increased the expression of Nox4 and aggravated the losses of circadian proteins, Clock, and Bmal1; in contrast, knockdown of circItm2b ameliorated these changes (Fig. 4F–I, Supplement Fig. 3A, B). In conclusion, these results demonstrated that circItm2b overexpression exacerbated the dysfunction of mitochondria, oxidative stress, and the losses of circadian rhythm proteins.

### CircItm2b directly interacts with Sirt1

**Fig. 5:**
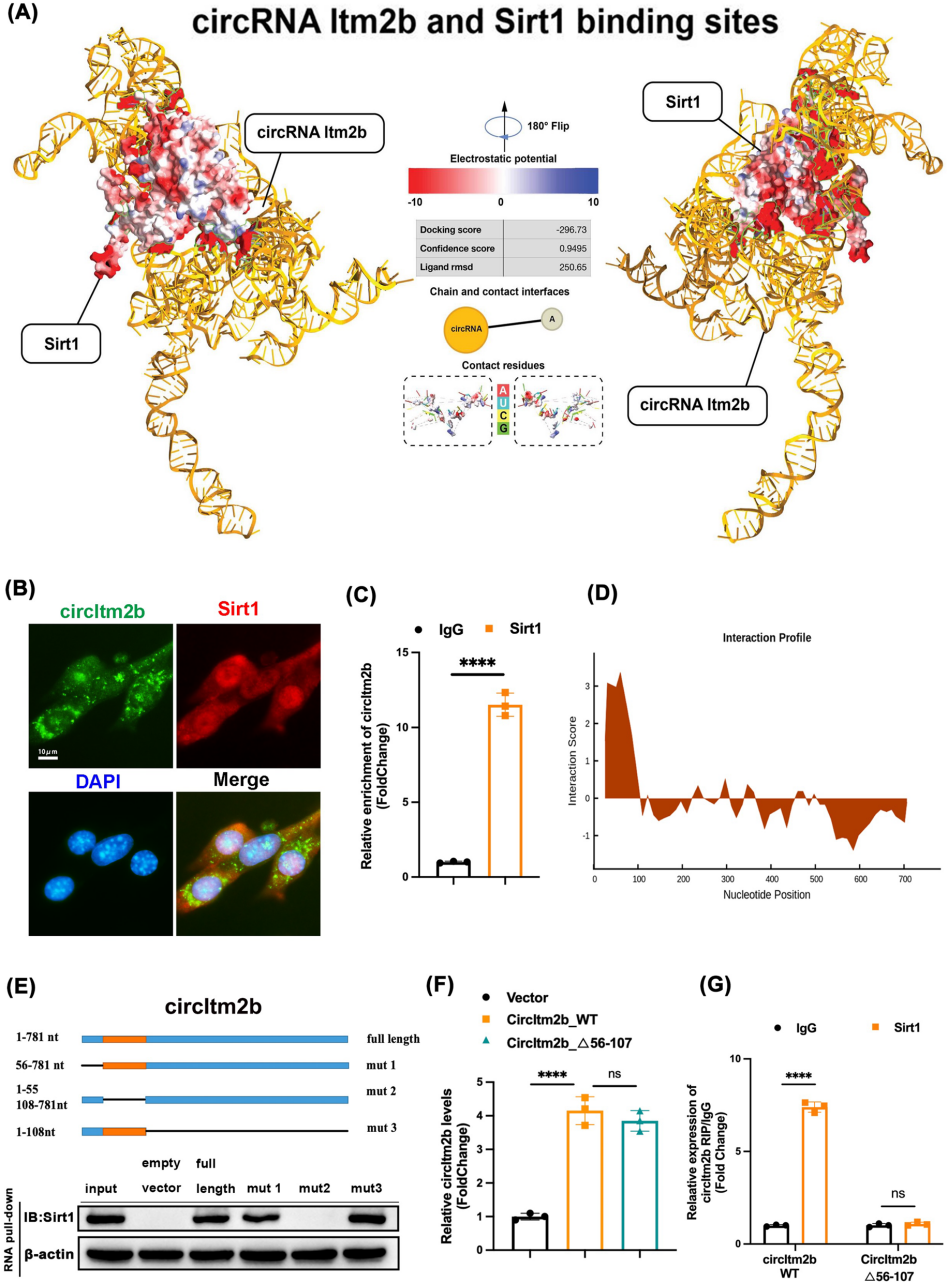
CircItm2b could bind the Sirt1. **A** The three-dimension docking structure diagram shows the circItm2b could bind the Sirt1, the docking score: − 296.73, the confidence score: 0.9495, the ligand rmsd: 250.65. **B** Representative FISH images reveal that circItm2b and Sirt1 are enriched in the cytoplasm of HT22 cells. **C** RIP results in HT22 cells confirmed that circItm2b could be enriched by Sirt1. IgG was employed as a negative control, **** p < 0.0001, one-way ANOVA followed by Tukey’s multiple comparisons test. **D** The catRAPID algorithm was used to predict the binding position of circItm2b to Sirt1. **E** Serial deletions of circItm2b were used to verify the regions of circItm2b that were required for Sirt1 in RNA pull-down assays. **F** The level of circItm2b was confirmed by qRT–PCR when mutated the binding position of circItm2b, circItm2b-WT vs. Vector, **** p < 0.0001; CircItm2b_∆56–107 vs. circItm2b-WT**,** ns not statistically significant, one-way ANOVA followed by Tukey’s multiple comparisons test. **G** RIP assay was performed to analyze the level of circItm2b after mutating the 56–107-nt region of circItm2b, circItm2b-WT + Sirt1 vs. circItm2b-WT + IgG, **** p < 0.0001, circItm2b-∆56–107 + Sirt1 vs. circItm2b-∆56–107 + IgG, ns not statistically significant, one-way ANOVA followed by Tukey’s multiple comparisons test. All data were represented as mean ± SD

Recently, significant attention has been drawn to the interaction between circRNAs and proteins [38]. The silent information regulator sirtuin 1 (Sirt1), which was involved in various biological processes, such as inflammation and redox homeostasis and may affect the sleep quality, caugut our attention [39, 40]. In our experiment, the expression effectively increased or decreased after injection of overexpressing or knockdown lentivirus (Supplement Fig. 3C, D). By treating RNA from mouse brain and HT22 cells with RNase R, we identified that the lentivirus affected circItm2b expression without impacting linear transcription (Supplement Fig. 3E–J). The sequence analysis of circItm2b and Sirt1 through HDDock (http://hdock.phys.hust.edu.cn/), and schematic illustration revealed that circItm2b could bind to Sirt1 (Fig. 5A). The co-localization of circItm2b and Sirt1 was confirmed by the FISH assay and immunofluorescence (Fig. 5B), and RIP results demonstrated the circItm2b could significantly enriched by Sirt1 (Fig. 5C). Subsequently, the catRAPID website (http://service.tartaglialab.com/page/catrapid_group) was employed to predict the potential binding region between circItm2b and Sirt1, the outcomes indicated that 56–107 nt region of circItm2b might necessary for Sirt1 interaction (Fig. 5D and Supplement Table 2). The next series of deletion assays revealed that the 56–107 nt region of circItm2b was essential for Sirt1 binding (Fig. 5E). Moreover, the level of circItm2b also increased after transfecting the mutation of the 56–107 nt region of circItm2b in cells (Fig. 5F), and RIP results demonstrated the reduced enrichment of circItm2b by Sirt1 antibodies following the mutation of the 56–107 nt region of circItm2b (Fig. 5G). These findings support that circItm2b may directly interact with Sirt1.

### CircItm2b regulates Nox4 expression through binding Sirt1

**Fig. 6:**
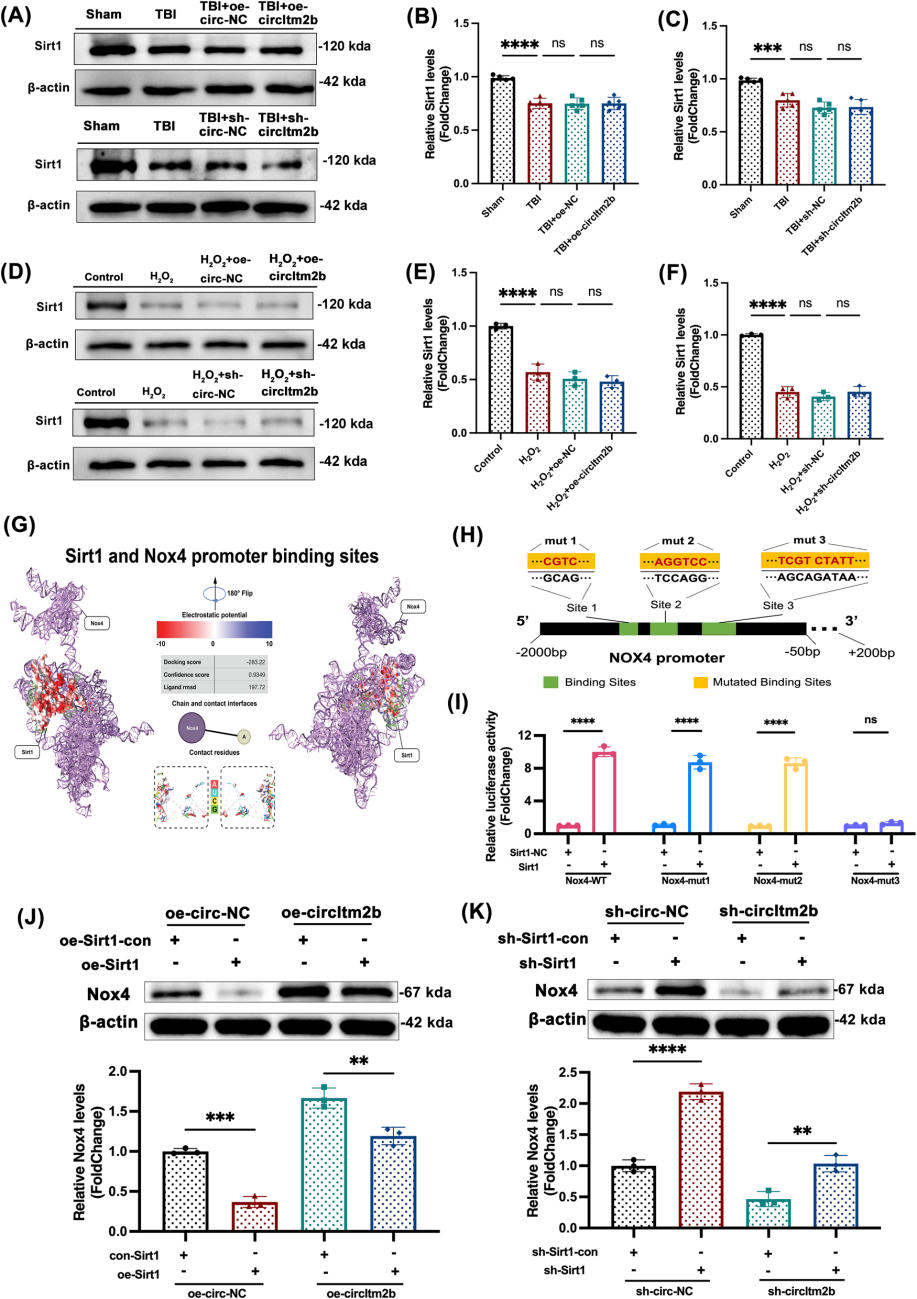
CircItm2b regulates Nox4 expression through binding Sirt1. **A–C** Western blot bands of Sirt1 after circItm2b overexpression or knockdown in mice. The expressions of Sirt1 are detected by western blotting after circItm2b overexpression in mice. n = 5 repetition, TBI vs. Sham group, **** p < 0.0001, TBI + oe-circ-NC vs. TBI, ns not statistically significant, TBI + oe-circItm2b vs. TBI + oe-circ-NC, ns not statistically significant, one-way ANOVA followed by Tukey’s multiple comparisons test**.** The expressions of Sirt1 are detected by western blotting after circItm2b knockdown in mice. n = 5 repetition, TBI vs. Sham group, *** p < 0.001, TBI + sh-circ-NC vs. TBI, ns not statistically significant, TBI + sh-circItm2b vs. TBI + sh-circ-NC, ns not statistically significant, one-way ANOVA followed by Tukey’s multiple comparisons test.** D–F** Western blot bands of Sirt1 after circItm2b overexpression or knockdown in cells. The expressions of Sirt1 are detected by western blotting after circItm2b overexpression in cells. n = 3 repetition, H_2_O_2_ (600 μmol/L) treated HT22 cells for 6 h. H_2_O_2_ vs. Control group, **** p < 0.0001; H_2_O_2_ + oe-circ-NC vs H_2_O_2_, ns not statistically significant; H_2_O_2_ + oe-circ vs. H_2_O_2_ + oe-circ-NC, ns not statistically significant, one-way ANOVA followed by Tukey’s multiple comparisons test**.** The expressions of Sirt1 are detected by western blotting after circItm2b knockdown in cells. n = 3 repetition, H_2_O_2_ (600 μmol/L) treated HT22 cells for 6 h. H_2_O_2_ vs. Control group, **** p < 0.0001; H_2_O_2_ + sh-circ-NC vs H_2_O_2_, ns not statistically significant; H_2_O_2_ + sh-circ vs. H_2_O_2_ + sh-circ-NC, ns not statistically significant, one-way ANOVA followed by Tukey’s multiple comparisons test. **G** The three-dimension docking structure diagram shows the Sirt1 could bind the Nox4 promoter, the docking score: −283.22, the confidence score: 0.9349, the ligand rmsd: 197.72. **H** Schematic illustration of the Nox4 promoter, the green block shows the binding sites and the base sequence, and the yellow block shows the mutated binding sites. **I** Luciferase assay shows the relative luciferase activity in 293 T cells after co-transfection of Nox4-WT/Nox4-mut1/Nox4-mut2/Nox4-mut3 and Sirt1/Sirt1-NC, n = 3 repetition, Nox4-WT + Sirt1 vs. Nox4-WT + Sirt1-NC, **** p < 0.0001; Nox4-mut1 + Sirt1 vs. Nox4-mut1 + Sirt1-NC, **** p < 0.0001; Nox4-mut2 + Sirt1 vs. Nox4-mut2 + Sirt1-NC, **** p < 0.0001; Nox4-mut3 + Sirt1 vs. Nox4-mut3 + Sirt1-NC, ns not statistically significant. One-way ANOVA followed by Tukey’s multiple comparisons test. **J **The expression of Nox4 after co-transfecting the oe-circ-NC + oe-Sirt1-con or oe-circ-NC + oe-Sirt1 or oe-circItm2b + oe-Sirt1-con or oe-circItm2b + oe-Sirt1. n = 3 repetition, oe-circ-NC + oe-Sirt1 vs. oe-circ-NC + oe-Sirt1-con, *** p < 0.001; oe-circItm2b + oe-Sirt1 vs. oe-circItm2b + oe-Sirt1-con, ** p < 0.01. **K** The expression of Nox4 after co-transfecting the sh-circ-NC + sh-Sirt1-con or sh-circ-NC + sh-Sirt1 or sh-circItm2b + sh-Sirt1-con or sh-circItm2b + sh-Sirt1. n = 3 repetition, sh-circ-NC + sh-Sirt1 vs. sh-circ-NC + sh-Sirt1-con, **** p < 0.0001; sh-circItm2b + sh-Sirt1 vs. sh-circItm2b + sh-Sirt1-con, ** p < 0.01. One-way ANOVA followed by Tukey’s multiple comparisons test. All data were represented as mean ± SD

The Sirt1 expression was detected, and the results showed the Sirt1 expression was decreased after TBI. However, transfection with overexpression or knockdown of circItm2b did not alter the level of Sirt1, either in vivo (Fig. 6A–C) or in vitro (Fig. 6D–F). Sirt1 has been reported as a multifunctional transcriptional regulator with NAD-dependent protein deacetylase activity [41]. We then employed HDDOCK to visualize the binding region of Sirt1 and the Nox4 promoter, as shown in the three-dimensional structure diagram in Fig. 6G. The exact binding region was identified after docking analysis between Sirt1 and the Nox4 promoter, revealing several continuous base segments (Site 1, Site 2 and Site 3) as potential binding sites on the Nox4 promoter. We first constructed the plasmid with point mutated the binding sites of the Nox4 promoter (Nox4 mut1, Nox4 mut2 and Nox4 mut3), and performed luciferase assays in 293 T cells to verify the interaction. The consequences indicated that the luciferase activity of Nox4 mut3 had no statistical significance, while the Nox4 mut1 or Nox4 mut2 showed significantly increased luciferase activity, which manifested the Site 3 was the possible binding sites of Nox4 promoter to bind the Sirt1(Fig. 6H, I). Finally, the overexpression of Sirt1 or knockdown of Sirt1 was co-transfected with the lentivirus of oe-circItm2b or the lentivirus of sh-circItm2b in HT22 cells. The blunted expression of Nox4 induced by oe-Sirt1 was ameliorated by the overexpression of circItm2b (Fig. 6J). Meanwhile, the elevated expression of Nox4 caused by sh-Sirt1 was reversed by the knockdown of circItm2b (Fig. 6K). As previously shown, circItm2b can directly bind the Sirt1. Taken together, these results indicated that Sirt1 acted as a mediator of circItm2b in regulating Nox4 expression.

### Sirt1 attenuates dysfunction of mitochondrial and oxidative stress-induced circadian rhythm protein losses by targeting Nox4

**Fig. 7:**
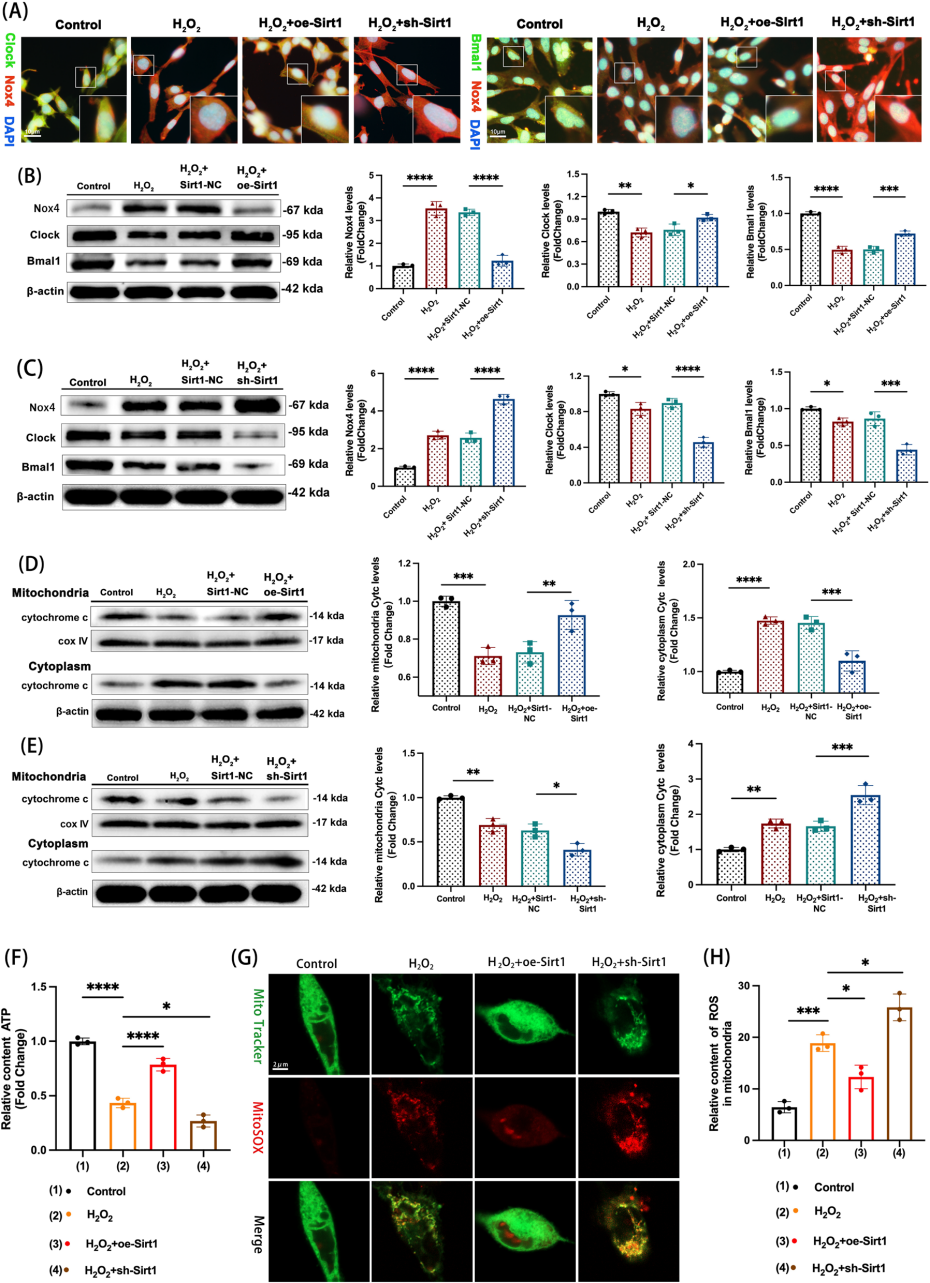
Sirt1 attenuates dysfunction of mitochondrial and oxidative stress-induced circadian rhythm protein losses by targeting Nox4. **A** The representative images of double immunofluorescence show the co-localization of Nox4, Clock, and Bmal1 in HT22 cells. **B** The expressions of Nox4, Clock, and Bmal1 are detected by western blotting in Control, H_2_O_2_, H_2_O_2_ + Sirt1-NC, and H_2_O_2_ + oe-Sirt1 groups. n = 3 repetitions. Nox4: H_2_O_2_ vs. Control group, **** p < 0.0001; H_2_O_2_ + oe-Sirt1 vs. H_2_O_2_ + Sirt1-NC, **** p < 0.0001. Clock: H_2_O_2_ vs. Control group, ** p < 0.01; H_2_O_2_ + oe-Sirt1 vs. H_2_O_2_ + Sirt1-NC, * p < 0.05. Bmal1: H_2_O_2_ vs. Control group, **** p < 0.0001; H_2_O_2_ + oe-Sirt1 vs. H_2_O_2_ + Sirt1-NC, *** p < 0.001. **C** The expressions of Nox4, Clock, and Bmal1 are detected by western blotting in Control, H_2_O_2_, H_2_O_2_ + Sirt1-NC, and H_2_O_2_ + sh-Sirt1 groups. n = 3 repetitions. Nox4: H_2_O_2_ vs. Control group, **** p < 0.0001; H_2_O_2_ + sh-Sirt1 vs. H_2_O_2_ + Sirt1-NC, **** p < 0.0001. Clock: H_2_O_2_ vs. Control group, * p < 0.05; H_2_O_2_ + sh- Sirt1 vs. H_2_O_2_ + Sirt1-NC, **** p < 0.0001. Bmal1: H_2_O_2_ vs. Control group, * p < 0.05; H_2_O_2_ + sh-Sirt1 vs. H_2_O_2_ + Sirt1-NC, *** p < 0.001. **D** Western blot analysis of the expression of cytochrome C in the mitochondria and the cytoplasm in the HT22 cells transduced with Sirt1-NC or oe-Sirt1 for 48 h and and finally treated with H_2_O_2_ (600 μmol/L) for 6 h, n = 3. Mitochondria: H_2_O_2_ vs. Control, *** p < 0.001, H_2_O_2_ + oe-Sirt1 vs. H_2_O_2_ + Sirt1-NC, ** p < 0.01; cytoplasm: H_2_O_2_ vs. control, **** p < 0.0001, H_2_O_2_ + oe-Sirt1 vs. H_2_O_2_ + Sirt1-NC, *** p < 0.001. One-way ANOVA followed by Tukey’s multiple comparisons test. **E** Western blot analysis of the expression of cytochrome C in the mitochondria and the cytoplasm in the HT22 cells transduced with Sirt1-NC or sh-Sirt1 for 48 h and finally treated with H_2_O_2_ (600 μmol/L) for 6 h, n = 3. Mitochondria: H_2_O_2_ vs. Control, ** p < 0.01, H_2_O_2_ + sh-Sirt1 vs. H_2_O_2_ + Sirt1-NC, * p < 0.05; cytoplasm: H_2_O_2_ vs. Control, ** p < 0.01, H_2_O_2_ + sh-Sirt1 vs. H_2_O_2_ + Sirt1-NC, *** p < 0.001. One-way ANOVA followed by Tukey’s multiple comparisons test. **F** The relative contents of ATP in HT22 cells are measured in Control, H_2_O_2_, H_2_O_2_ + oe-Sirt1, and H_2_O_2_ + sh-Sirt1 groups. n = 3, H_2_O_2_ vs. Control group, **** p < 0.0001; H_2_O_2_ + oe-Sirt1 vs. H_2_O_2_, **** p < 0.0001; H_2_O_2_ + sh-Sirt1 vs. H_2_O_2_, * p < 0.05. One-way ANOVA followed by Tukey’s multiple comparisons test. **G** Representative images of fluorescent staining of MitoSox for mitochondrial ROS in HT22 cells in the group of Control, H_2_O_2_, H_2_O_2_ + oe-Sirt1, and H_2_O_2_ + sh-Sirt1. **H** The relative content of ROS in mitochondria, n = 3, H_2_O_2_ vs. Control, *** p < 0.001; H_2_O_2_ + oe-Sirt1 vs. H_2_O_2_, * p < 0.05; H_2_O_2_ + sh-Sirt1 vs. H_2_O_2_, * p < 0.05. One-way ANOVA followed by Tukey’s multiple comparisons test. All data were represented as mean ± SD

The oe-Sirt1 and the sh-Sirt1 were transfected into the HT22 cells for 48 h and followed by treatment with the H_2_O_2_ (600 μM/L) to verify whether the Sirt1 regulated the Nox4. The immunofluorescent staining results revealed the co-localization of the Nox4, Clock, and Bmal1 (Fig. 7A, Supplement Fig. 3K–L), and expression levels were detected by Western blotting. Transfection of oe-Sirt1 into H_2_O_2_-treated HT22 cells alleviated the mitochondrial oxidative stress-induced loss of circadian rhythm proteins, significantly reducing Nox4 expression and increasing Clock and Bmal1 levels (Fig. 7B). Accordingly, transfecting the sh-Sirt1 into H_2_O_2_-treated HT22 cells increased the Nox4 levels and down-regulated Clock and Bmal1 expression (Fig. 7C). The expression of cytochrome C increased in mitochondria and translocated less to the cytoplasm when transfected the oe-Sirt1 into H_2_O_2_-treated HT22 cells, while the reverse was observed with sh-Sirt1 (Fig. 7D, E). Furthermore, oe-Sirt1 increased ATP content in H_2_O_2_-treated cells ( Fig. 7F). Fluorescent staining of MitoSox demonstrated that oe-Sirt1 mitigated mitochondrial oxidative stress, while sh-Sirt1 exacerbated it (Fig. 7G). The relative content of ROS in mitochondria was reduced after oe-Sirt1 transfection and increased after sh-Sirt1 transfection (Fig. 7H). Overall, the results suggested that the Sirt1 attenuates the dysfunction of mitochondrial and oxidative stress-induced circadian rhythm protein losses by targeting Nox4.

### CircItm2b/Sirt1/Nox4 signaling axis regulated dysfunction of mitochondria and oxidative stress after TBI

**Fig. 8:**
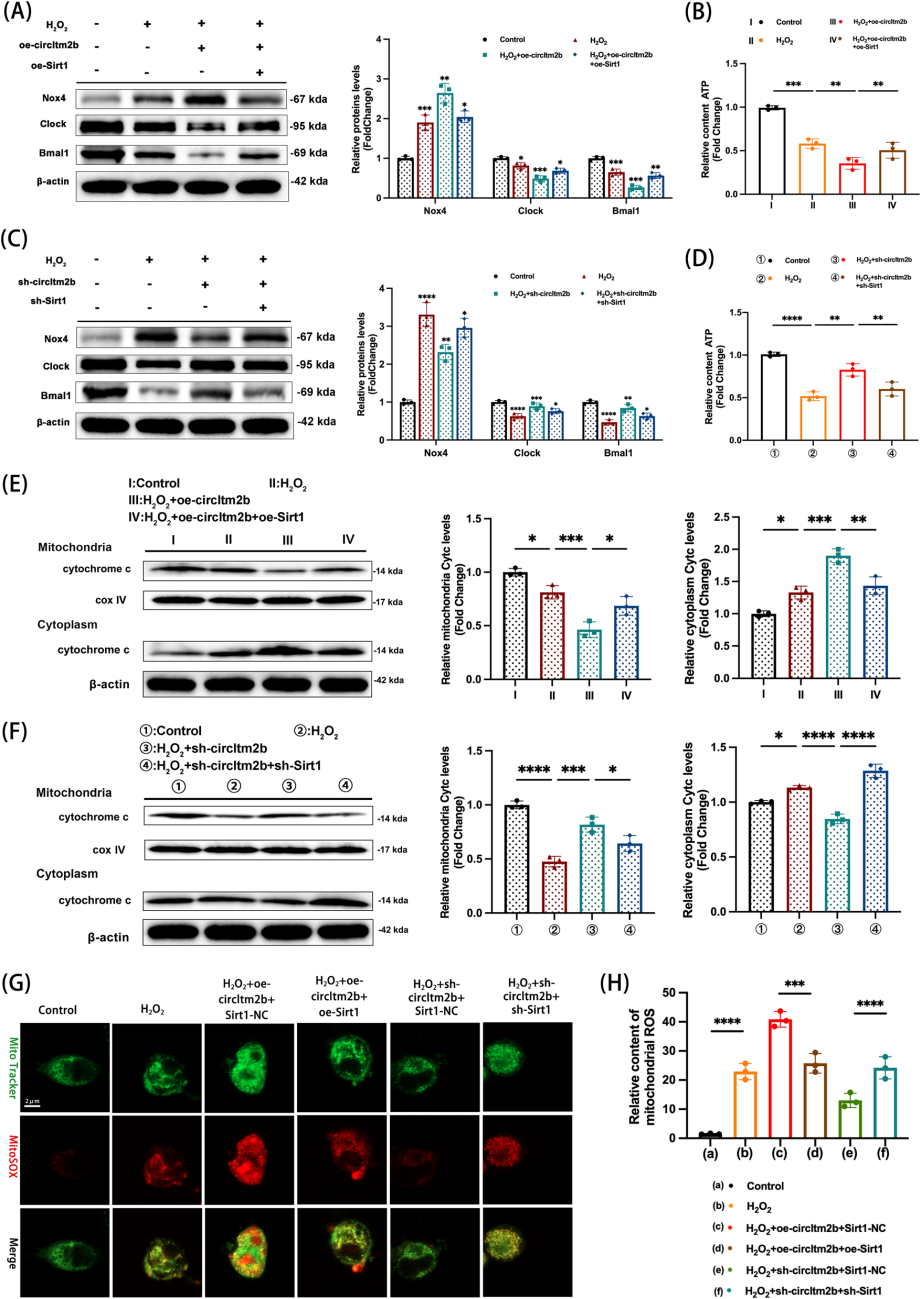
CircItm2b/ Sirt1/Nox4 signaling axis regulated mitochondrial dysfunction and oxidative stress after TBI. **A** The expressions of Nox4, Clock and Bmal1 are detected by western blotting in HT22 cells transduced with lentivirus of oe-circItm2b for 7 days, then transduced with oe-Sirt1 for 48 h, and finally, H_2_O_2_ (600 μmol/L) treated HT22 cells for 6 h. Nox4: H_2_O_2_ vs. Control group, *** p < 0.001; H_2_O_2_ + oe-circItm2b vs H_2_O_2_, ** p < 0.01; H_2_O_2_ + oe-circItm2b + oe-Sirt1 vs. H_2_O_2_ + oe-circItm2b, * p < 0.05. Clock: H_2_O_2_ vs. Control group, * p < 0.05; H_2_O_2_ + oe-circItm2b vs H_2_O_2_, *** p < 0.001; H_2_O_2_ + oe-circItm2b + oe-Sirt1 vs. H_2_O_2_ + oe-circItm2b, * p < 0.05. Bmal1: H_2_O_2_ vs. Control group, *** p < 0.001; H_2_O_2_ + oe-circItm2b vs H_2_O_2_, *** p < 0.001; H_2_O_2_ + oe-circItm2b + oe-Sirt1 vs. H_2_O_2_ + oe-circItm2b, ** p < 0.01. One-way ANOVA followed by Tukey’s multiple comparisons test. **B** The relative contents of ATP in HT22 cells are measured in Control, H_2_O_2_, H_2_O_2_ + oe-circItm2b, and H_2_O_2_ + oe-circItm2b + oe-Sirt1 groups. n = 3, H_2_O_2_ vs. Control group, *** p < 0.001; H_2_O_2_ + oe-circItm2b vs. H_2_O_2_, ** p < 0.01; H_2_O_2_ + oe-circItm2b + oe-Sirt1 vs. H_2_O_2_ + oe-circItm2b, ** p < 0.01. One-way ANOVA followed by Tukey’s multiple comparisons test. **C** The expressions of Nox4, Clock and Bmal1 are detected by western blotting in HT22 cells transduced with lentivirus of sh-circItm2b for 7 days, then transduced with sh-Sirt1 for 48 h, and finally, H_2_O_2_ (600 μmol/L) treated HT22 cells for 6 h. Nox4: H_2_O_2_ vs. Control group, **** p < 0.0001; H_2_O_2_ + sh-circItm2b vs H_2_O_2_, ** p < 0.01; H_2_O_2_ + sh-circItm2b + sh-Sirt1 vs. H_2_O_2_ + sh-circItm2b, * p < 0.05. Clock: H_2_O_2_ vs. Control group, **** p < 0.0001; H_2_O_2_ + sh-circItm2b vs H_2_O_2_, *** p < 0.001; H_2_O_2_ + sh-circItm2b + sh-Sirt1 vs. H_2_O_2_ + sh-circItm2b, * p < 0.05. Bmal1: H_2_O_2_ vs. Control group, **** p < 0.0001; H_2_O_2_ + sh-circItm2b vs H_2_O_2_, ** p < 0.01; H_2_O_2_ + sh-circItm2b + sh-Sirt1 vs. H_2_O_2_ + sh-circItm2b, * p < 0.05. One-way ANOVA followed by Tukey’s multiple comparisons test. **D** The relative contents of ATP in HT22 cells are measured in Control, H_2_O_2_, H_2_O_2_ + sh-circItm2b, and H_2_O_2_ + sh-circItm2b + sh-Sirt1 groups. n = 3, H_2_O_2_ vs. Control group, **** p < 0.0001; H_2_O_2_ + sh-circItm2b vs. H_2_O_2_, ** p < 0.01; H_2_O_2_ + sh-circItm2b + sh-Sirt1 vs. H_2_O_2_ + sh-circItm2b, ** p < 0.01. One-way ANOVA followed by Tukey’s multiple comparisons test. **E** Western blot analysis of cytochrome C in HT22 cells transduced with oe-circItm2b lentivirus cells for 7 days, then transduced with oe-Sirt1 for 48 h, finally treated with H_2_O_2_ (600 μmol/L) for 6 h. n = 3. Mitochondria: H_2_O_2_ vs. Control, * p < 0.05; H_2_O_2_ + oe-circItm2b vs. H_2_O_2_, *** p < 0.001; H_2_O_2_ + oe-circItm2b + oe-Sirt1 vs. H_2_O_2_ + oe-circItm2b, * p < 0.05; cytoplasm: H_2_O_2_ vs. Control, * p < 0.05; H_2_O_2_ + oe-circItm2b vs. H_2_O_2_, *** p < 0.001; H_2_O_2_ + oe-circItm2b + oe-Sirt1 vs. H_2_O_2_ + oe-circItm2b, ** p < 0.01. One-way ANOVA followed by Tukey’s multiple comparisons test. **F** Western blot analysis of cytochrome C in HT22 cells transduced with sh-circItm2b lentivirus cells for 7 days, then transduced with sh-Sirt1 for 48 h, finally treated with H_2_O_2_ (600 μmol/L) for 6 h. n = 3. Mitochondria: H_2_O_2_ vs. Control, **** p < 0.0001; H_2_O_2_ + sh-circItm2b vs. H_2_O_2_, *** p < 0.001; H_2_O_2_ + sh-circItm2b + sh-Sirt1 vs. H_2_O_2_ + sh-circItm2b, * p < 0.05; cytoplasm: H_2_O_2_ vs. Control, * p < 0.05; H_2_O_2_ + sh-circItm2b vs. H_2_O_2_, **** p < 0.0001; H_2_O_2_ + sh-circItm2b + sh-Sirt1 vs. H_2_O_2_ + sh-circItm2b, **** p < 0.0001. One-way ANOVA followed by Tukey’s multiple comparisons test. **G** Representative images of fluorescent staining of MitoSox for mitochondrial ROS in HT22 cells in the group of Control, H_2_O_2_, H_2_O_2_ + oe-circItm2b + Sirt1-NC, H_2_O_2_ + oe-circItm2b + oe-Sirt1, H_2_O_2_ + sh-circItm2b + Sirt1-NC, H_2_O_2_ + sh-circItm2b + sh-Sirt1. **H** The relative content of ROS in mitochondria, n = 3, H_2_O_2_ vs. Control, **** p < 0.0001; H_2_O_2_ + oe-circItm2b + oe-Sirt1 vs. H_2_O_2_ + oe-circItm2b + oe-Sirt1-NC, *** p < 0.001; H_2_O_2_ + sh-circItm2b + sh-Sirt1 vs. H_2_O_2_ + sh-circItm2b + Sirt1-NC, **** p < 0.0001. One-way ANOVA followed by Tukey’s multiple comparisons test. All data were represented as mean ± SD

The rescue experiments were executed to further confirm whether the circItm2b influenced the mitochondrial function through Sirt1/Nox4 axis. Our consequences demonstrated the overexpression of circItm2b aggravated the mitochondrial oxidative stress-induced circadian rhythm protein losses. Specifically, we found that elevated Nox4 expression and decreased Clock and Bmal1 levels, which are considered negative impacts, were blunted by co-transfection of oe-Sirt1 (Fig. 8A). Additionally, ATP content showed a reversed trend after co-transfection of oe-circItm2b and oe-Sirt1 (Fig. 8B). When sh-circItm2b was transfected alone, the mitochondrial oxidative stress-induced circadian rhythm protein losses were alleviated, the levels of Nox4 attenuated, and the Clock and Bmal1 expression increased, however, co-transfection of the sh-Sirt1 reversely altered these effects (Fig. 8C), the similar trend was also observed for relative ATP content (Fig. 8D). Subsequently, the results showed the circItm2b overexpression alone aggravated cytochrome C translocated from the mitochondria to the cytoplasm, while co-transfection with oe-Sirt1 rescued this process (Fig. 8E). In addition, circItm2b knockdown alone reduced cytochrome C translocation from the mitochondria to the cytoplasm, while co-transfected with sh-Sirt1 aggravated this process (Fig. 8F). The immunofluorescent results and the measurements of mitochondrial ROS revealed that the overexpression of circItm2b aggravated the mitochondrial oxidative stress, however, transfection with the oe-Sirt1 rescued these effects. Transfection with sh-circItm2b alleviated the mitochondrial oxidative stress, while co-transfection with sh-Sirt1 reversed these effects of sh-circItm2b (Fig. 8G-H). Collectively, these results identify that knockdown of circItm2b alleviates mitochondrial dysfunction and enhances antioxidant stress capacity through the circItm2b/Sirt1/Nox4 axis.

## Discussion

**Fig. 9:**
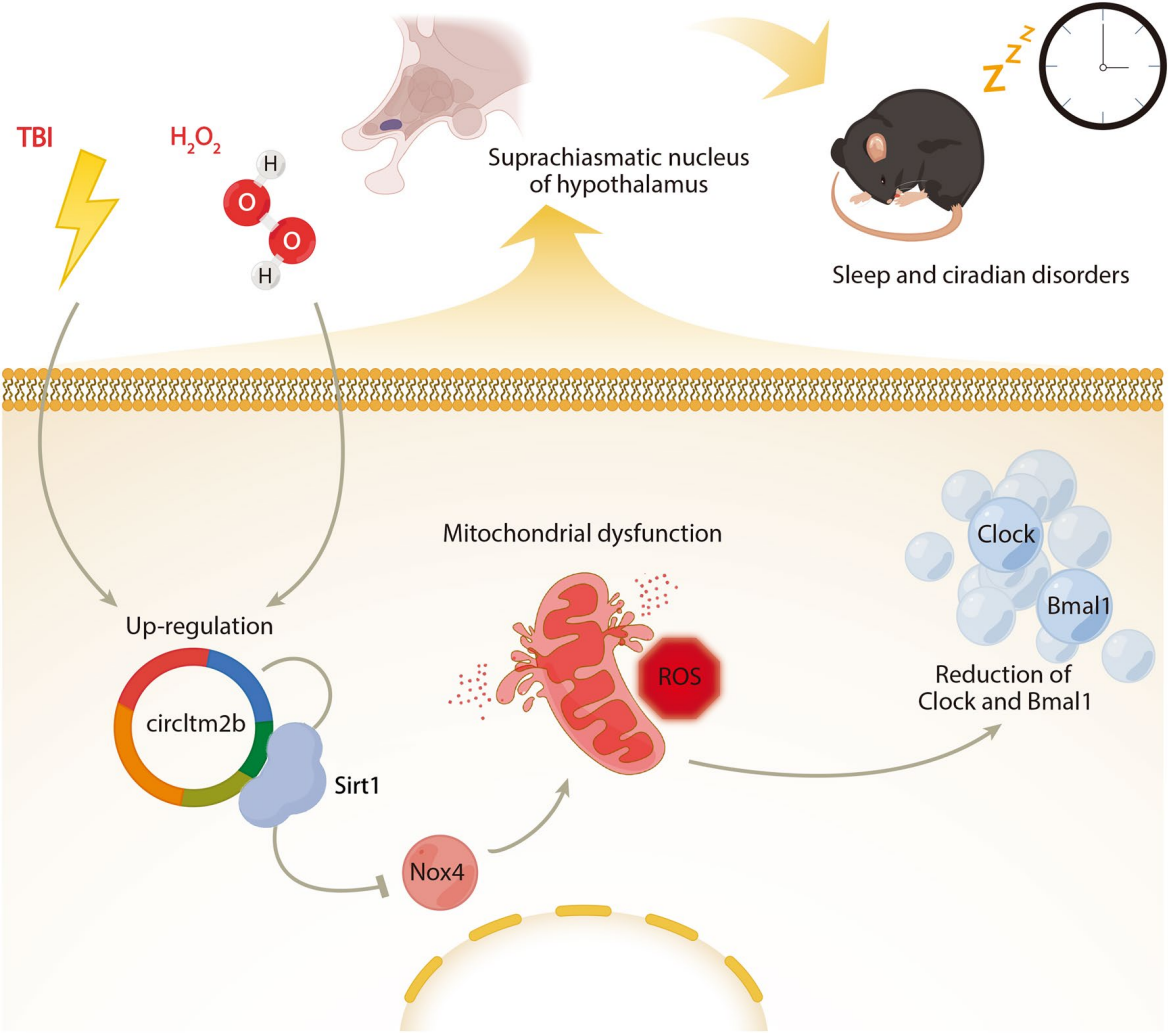
The mechanism diagram demonstrates that circtm2b exacerbates mitochondrial oxidative stress and causes sleep disorders. The mechanism diagram was plotted by Figdraw and Biorender. TBI or H_2_O_2_ intervention leads to upregulation of circItm2b, mitochondrial dysfunction, and oxidative stress. The increased circItm2b binding the Sirt1, which increased the Nox4 expression. The upregulated Nox4 aggravates mitochondrial oxidative stress. Mitochondrial dysfunction and losses of Clock and Bmal1 disrupt the circadian rhythm in the suprachiasmatic nucleus of the hypothalamus. CircItm2b is used as a new target for sleep disorders after TBI

To the best of our knowledge, this is the first study providing evidence that circItm2b was associated with sleep disorders after TBI. In this study, we found that circItm2b, which is elevated after TBI, might serve as a diagnostic biomarker for sleep and circadian disorders induced by TBI. Firstly, the RNA-seq verified the circRNAs with differential expression after TBI, and the mmu_circ_0005429 (circItm2b) was confirmed as our target gene. Next, the homology analysis revealed that the mmu_circ_0005429 and the hsa_circ_0006620 (linear transcript *ITM2B*) have identity reached 89.2%, and the increased expression of hsa_circ_0006620 in the serum of TBI patients was related to sleep disorders. These two circRNAs, mmu_circ_0005429 and hsa_circ_0006620, might be the potential biomarkers of sleep disorders after TBI. A complex interdependence between sleep disturbance and oxidative stress has been previously reported [6]. The behavioral experiments of mice further identified that the overexpression of mmu_circ_0005429 was correlated with sleep disorders after TBI, and the subsequent in vivo results further demonstrated that it could exacerbate the oxidation states and lead to circadian protein losses, potentially contributing to sleep disorders after TBI. To explore the molecular mechanism, HT22 cells were cultured and treated with H_2_O_2_ to construct an oxidative stress model. The consequences finally suggested that the knockdown of circItm2b alleviated mitochondrial oxidative stress-induced losses of circadian rhythm proteins through the circItm2b/ Sirt1/Nox4 signaling axis (Fig. 9).

Up to now, limited studies have explored the relationship between circRNAs and sleep or circadian disorders. A recent study suggested that Cdr1as circRNA might be a new gene linked to the light–dark cycle entrainment in the SCN [19]. However, hardly any studies have indicated the role of circRNA in sleep disturbance in TBI. A subset of circRNAs has been found to be highly enriched in the brain [42], and some structures of the cerebra have been strongly correlated with memory, sleep, psychiatric diseases, and neurodegeneration [43–45], due to these conditions often parallelly occurring. In the context of TBI, the dysregulation of circRNAs in the brain might link the sleep disorder to brain injury. In our research, sleep scales and EEG were used to diagnose sleep disturbances as previously reported [46], we found that the sleep scale results from TBI patients were correlated with the overexpression of circItm2b. EEG consequences in TBI patients further verified the disruption of sleep architecture after TBI and pointed out that the hsa_circ_0006620 might be a potential biomarker in sleep disturbance after TBI. EEG results in mice showed a decrease in delta waves at 14 and 30 days post-TBI, and inhibition of circItm2b could improve the reduction in delta waves. Our results were consistent with previous findings on EEG characteristics in patients with mTBI and insomnia, namely, where delta energy reduction was observed in sleep duration in TBI patients [47, 48]. Herein, our study confirms that circItm2b is involved in sleep disturbances following TBI.

Mitochondrial dysfunction and oxidative stress are critical pathophysiological processes in secondary brain injury following TBI [49]. It has been established that mitochondrial oxidative stress is intricately linked to sleep disturbances in a bidirectional manner [6, 10, 50]. Studies have shown that circadian disturbances after TBI can disrupt the sleep–wake cycle [2, 4], which inhibited neurogenesis after TBI [51]. Additionally, many circRNAs have been found to be closely associated with oxidative stress in neurological diseases [52], however, few studied have explored the relationship between the oxidative stress, circRNA alternation and sleep and circadian disorders. Our data showed that overexpression of circItm2b elevated the mitochondrial ROS level, and decreased the content of ATP, furthermore, it increased the expression of Nox4 and reduced the level of Clock and Bmal1, which in keeping with the research of Gaudet et al., who reported that the circadian system in rats was disrupted following spinal cord injury (SCI) due to a reduction in the expression of the core circadian rhythm proteins, Clock and Bmal1 [53]. Nevertheless, notably, Niu et al. indicated that the Clock expression was lower in the sleep disorder group in oral mucosal and mononuclear cells of TBI patients, and the Bmal1 expression had no significance compared to the control group [54]. It seems different from our consequences; we hypothesize that this discrepancy may be due to differences in the sample types and time points used in their study. Together, these results demonstrated that sleep or circadian disorders caused by TBI might attribute to the circItm2b upregulation-induced mitochondrial oxidative stress and the reduction of circadian rhythm proteins.

The most common mechanism of circRNA function is as a molecular sponge for miRNA, however, the interaction with proteins of circRNAs recently received attention. CircSLC4A7 was reported to interact with the HSP90 to accelerates stemness and progress of gastric cancer [38], and the circMETTL9 was researched as a regulator of neuroinflammation of TBI by complexing with the SND1[55]. In our study, we identified Sirt1 as a potential binding partner for circItm2b, as Sirt1 has been shown to have antioxidant properties and may regulate Nox4 expression [56, 57]. Thus, the binding analysis was performed and the followed pull-down and RIP assay to validate the binding site region of Sirt1 and circItm2b. However, although Sirt1 has been implicated in modulating Nox4 levels to alleviate oxidative stress, the exact pattern of interaction has not been fully elucidated. The Sirt1 was recorded it has transcriptional activity [41], which led us to perform binding of the Nox4 promoter with Sirt1, and further luciferase activity experiments demonstrated the two potential binding sites of Nox4 promoter with Sirt1. Then, the next outcomes suggested the Nox4 expression could be altered by the interaction between circItm2b and Sirt1. Moreover, overexpression or knockdown of Sirt1 verified that it could regulate the expression of Nox4, Clock, and Bmal1, influencing the redox status. These results are consistent with previous studies indicating that Sirt1 activation plays a critical role in regulating circadian rhythm genes, such as Clock and Bmal1, which are essential for stabilizing the sleep–wake cycle [58]. Given that circItm2b acts on Nox4 potentially through the regulation of Sirt1, we performed the rescue experiment and found that circItm2b attenuated the mitochondrial oxidative stress and promoted the capacity of antioxidant stress after TBI through circItm2b/Sirt1/Nox4 signaling pathway.

Above all, our findings for the first time suggested the circItm2b up-regulation after TBI might relate to the subsequent sleep disorders. Additionally, the molecular mechanism underlying the action of circItm2b has been elucidated. This study identifies a novel target for sleep disorders induced by TBI, which could serve as an effective therapeutic biomarker for TBI.

## Supplementary Information

Additional file 1: Figure S1. Relative circRNAs expression in TBI mice and H_2_O_2_-treated HT22 cells and the alternation of mRNA or fluorescence intensity and EEG α/β energy density in TBI patients. **(A)** Relative top 5 up-regulated circRNAs expression levels detected by qRT-PCR in TBI mice brain. n = 5. TBI vs. Sham, **** p < 0.0001. **(B)** Relative top 5 up-regulated circRNAs expression levels detected by qRT-PCR in HT22 cells treated by H_2_O_2_. n = 3. H_2_O_2_ vs. Control, ** p < 0.01, *** p < 0.001. **(C)** Relative *CLOCK*, *BMAL1* and *NOX4* mRNA expression levels detected by qRT-PCR in TBI patients brain specimen. TBI patients n = 6, controls n = 3. TBI vs. Control, *** p < 0.001, **** p < 0.0001. **(D-E) **Relative fluorescence intensity in double immunofluorescence show the intensity of NOX4, CLOCK or BMAL1 in patients with acute TBI. TBI patients n = 6, controls n = 3. TBI vs. Control, ** p < 0.01, *** p < 0.001, **** p < 0.0001. One-way ANOVA followed by Tukey’s multiple comparisons test. **(F-G)** The correlation analysis between alpha, beta energy density in sleep wake cycle and expression of hsa_circ_0006620 in the serum of TBI patients are shown. Grey points and lines represent the normal group; green points and lines represent the mild TBI group; red points and lines represent the moderate TBI group. All data were represented as mean ± SD.

Additional file 2: Figure S2. Relative EEG α/β energy density changes in TBI mice on 14d and 30d. **(A-B) ** Relative alpha, energy density in the ipsilesional and contralesional cortex at 14d after TBI. n = 5 mice per group. **(C-D) **Relative beta energy density in the ipsilesional and contralesional cortex at 14d after TBI. n = 5 mice per group. **(E-F)** Relative alpha energy density in the ipsilesional and contralesional cortex at 30d after TBI. n = 5 mice per group. **(G-H)** Relative beta energy density in the ipsilesional and contralesional cortex at 30d after TBI. n = 5 mice per group. One-way ANOVA followed by Tukey’s multiple comparisons test. All data were represented as mean ± SD

Additional file 3: Figure S3. The relative fluorescence intensity alternations and identification of circular structure of circItm2b. **(A) **Relative fluorescence intensity in double immunofluorescence shows the intensity of Nox4 and Clock in TBI mice brain. n = 5, Nox4: TBI vs. Sham, **** p < 0.0001, TBI + oe-circItm2b vs. TBI, * p < 0.01, TBI + sh-circItm2b vs. TBI, * p < 0.01. Clock: TBI vs. Sham, **** p < 0.0001, TBI + oe-circItm2b vs. TBI, ** p < 0.01, TBI + sh-circItm2b vs. TBI, **** p < 0.0001. **(B)** Relative fluorescence intensity in double immunofluorescence shows the intensity of Nox4 and Bmal1 in TBI mice brain. n = 5, Nox4: TBI vs. Sham, *** p < 0.001, TBI + oe-circItm2b vs. TBI, *** p < 0.001, TBI + sh-circItm2b vs. TBI, ** p < 0.01. Bmal1: TBI vs. Sham, **** p < 0.0001, TBI + oe-circItm2b vs. TBI, ** p < 0.01, TBI + sh-circItm2b vs. TBI, ** p < 0.01. **(C) **Relative circItm2b expression level detected by qRT-PCR in HT22 cells after the transduction of overexpression lentivirus for 7 days. n = 3 replications, *** p < 0.001, two-tailed t-test. **(D) **Relative circItm2b expression level detected by qRT-PCR in HT22 cells after the transduction of knockdown lentivirus for 7 days. n = 3 replications, *** p < 0.001, two-tailed t-test. **(E) **Relative linear Itm2b mRNA expression detected by qRT-PCR in mice after oe-circItm2b lentivirus transduction by Rnase R. n = 5 replications. Rnase R + oe-circ-NC vs. Control + oe-circ-NC, **** p < 0.0001, Rnase R + oe-circItm2b vs. Control + oe-circItm2b, #### p < 0.0001. Two-way ANOVA followed by Tukey’s multiple comparisons test. **(F) **Relative linear Itm2b mRNA expression detected by qRT-PCR in mice after sh-circItm2b lentivirus transduction by Rnase R. n = 5 replications. Rnase R + sh-circ-NC vs. Control + sh-circ-NC, **** p < 0.0001, Rnase R + sh-circItm2b vs. Control + sh-circItm2b, #### p < 0.0001. Two-way ANOVA followed by Tukey’s multiple comparisons test. **(G) **Relative linear Itm2b mRNA expression detected by qRT-PCR in HT22 cells after oe-circItm2b lentivirus transduction by Rnase R. n = 3 replications. Rnase R + oe-circ-NC vs. Control + oe-circ-NC, **** p < 0.0001, Rnase R + oe-circItm2b vs. Control + oe-circItm2b, #### p < 0.0001. Two-way ANOVA followed by Tukey’s multiple comparisons test. **(H) **Relative circItm2b expression detected by qRT-PCR in HT22 cells after oe-circItm2b lentivirus transduction by Rnase R. n = 3 replications. Control + oe-circItm2b vs. Control + oe-circ-NC, **** p < 0.0001; Rnase R + oe-circItm2b vs. Rnase R + oe-circ-NC, **** p < 0.0001. Two-way ANOVA followed by Tukey’s multiple comparisons test.** (I)** Relative linear Itm2b mRNA expression detected by qRT-PCR in HT22 cells after sh-circItm2b lentivirus transduction by Rnase R. n = 3 replications. Rnase R + sh-circ-NC vs. Control + sh-circ-NC, **** p < 0.0001, Rnase R + sh-circItm2b vs. Control + sh-circItm2b, #### p < 0.0001. Two-way ANOVA followed by Tukey’s multiple comparisons test. **(J) **Relative circItm2b expression detected by qRT-PCR in HT22 cells after sh-circItm2b lentivirus transduction by Rnase R. n = 3 replications. Control + sh-circItm2b vs. Control + sh-circ-NC, **** p < 0.0001; Rnase R + sh-circItm2b vs. Rnase R + sh-circ-NC, *** p < 0.001. Two-way ANOVA followed by Tukey’s multiple comparisons test. **(K) **Relative fluorescence intensity in double immunofluorescence shows the intensity of Nox4 and Clock in H_2_O_2_ treated HT22 cells. n = 3, Nox4: H_2_O_2_ vs. Control, **** p < 0.0001, H_2_O_2_ + oe-Sirt1 vs. H_2_O_2_, * p < 0.05, H_2_O_2_ + sh-Sirt1 vs. H_2_O_2_, ** p < 0.01. Clock: H_2_O_2_ vs. Control, *** p < 0.001, H_2_O_2_ + oe-Sirt1 vs. H_2_O_2_, * p < 0.05, H_2_O_2_ + sh-Sirt1 vs. H_2_O_2_, * p < 0.05. **(L)** Relative fluorescence intensity in double immunofluorescence shows the intensity of Nox4 and Bmal1 in H_2_O_2_ treated HT22 cells. n = 3, Nox4: H_2_O_2_ vs. Control, *** p < 0.001, H_2_O_2_ + oe-Sirt1 vs. H_2_O_2_, ** p < 0.01, H_2_O_2_ + sh-Sirt1 vs. H_2_O_2_, * p < 0.05. Bmal1: H_2_O_2_ vs. Control, **** p < 0.0001, H_2_O_2_ + oe-Sirt1 vs. H_2_O_2_, * p < 0.05, H_2_O_2_ + sh-Sirt1 vs. H_2_O_2_, * p < 0.05. One-way ANOVA followed by Tukey’s multiple comparisons test. All data were represented as mean ± SD.

Additional file 4: Table S1. Clinical data from control and TBI cases.

Additional file 5: Table S2. The binding score between circItm2b and SIRT1.

## Data Availability

No dataset was generated or analyzed during this study.

## Abbreviations

SD: Sleep disorders
TBI: Traumatic brain injury
SOD: Superoxide dismutase
CircRNAs: Circular RNAs
GCS: Glasgow coma scale
EEG: Electroencephalography
PSQI: Pittsburgh sleep quality index
AIS: Athens insomnia scale
CCI: Controlled cortical impact
mNSS: Modified neurological severity score test
RIP: RNA immunoprecipitation
ROC: Receiver operating characteristic
AUC: Area under curve
NOX: NADPH oxidase
Clock: Circadian locomotor output cycles kaput
Bmal1: Brain and muscle arnt-like 1
cDNA: Complementary DNA
gDNA: Genomic DNA
Sirt1: Silent information regulator sirtuin 1
SCI: Spinal cord injury
