# Correction: CircRNA Itm2b induces oxidative stress via the interaction with Sirt1-Nox4 to aggravate sleep disturbances after traumatic brain injury

**DOI:** 10.1186/s13578-025-01413-x

**Published:** 2025-10-09

**Authors:** Jiayuanyuan Fu, Mengran Du, Biying Wu, Chenrui Wu, Xin Li, Weilin Tan, Xuekang Huang, Ziyu Zhu, Jie Zhang, Zheng Bu Liao

**Affiliations:** 1 Department of Neurosurgery, The First Affiliated Hospital of Chongqing Medical University, No. 1 Youyi Road, Yuanjiagang, Yuzhong District, Chongqing, 400016 China; 2 Department of Neurosurgery, The Second Affiliated Hospital of Chongqing Medical University, Yuzhong District, Chongqing, 400010 China

**Correction: Cell & Bioscience (2025) 15:21** 10.1186/s13578-025-01353-6

In this article [1], the author would like to correct the mistake in Fig. 6I. The corrected and incorrected versions of Figure 6 are given below. The description of Fig. 6I in page 13 should be: The consequences indicated that the luciferase activity of Nox4 mut3 showed significantly increased，while the luciferase activity of Nox4 mut1 and Nox4 mut2 had no statistical significance, which manifested the Site 3 was the possible binding site of Nox4 promoter to bind the Sirt1 (Fig. 6H, I). Besides, several clerical inaccuracies that need correction. 1) In the figure legend of Fig.1 (page 6), the "Fold Change= 1" need to change to "Fold Change= ±1". 2) In Fig. 3I, the "Ipsi-Deta" need to change to "Ipsi-Delta", and "Control-Deta" need to change to "Control-Delta". 3) In page 21 line 1 of the text in left panel, "the two potential binding sites of Nox4 promoter with Sirt1." need to change to "the potential binding site of Nox4 promoter with Sirt1."

Incorrect Fig. 6
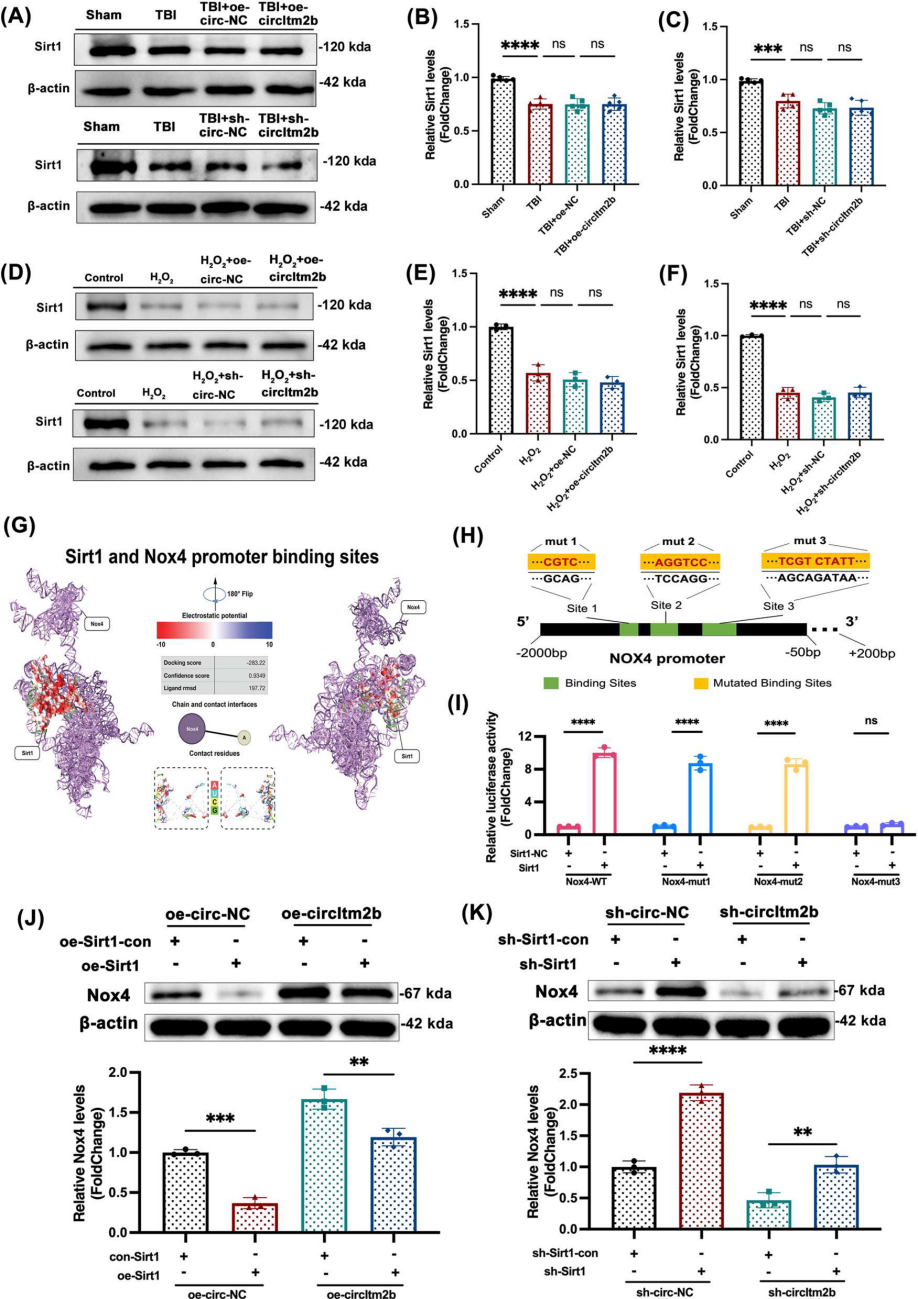


Correct Fig. 6

**Fig. 6:**
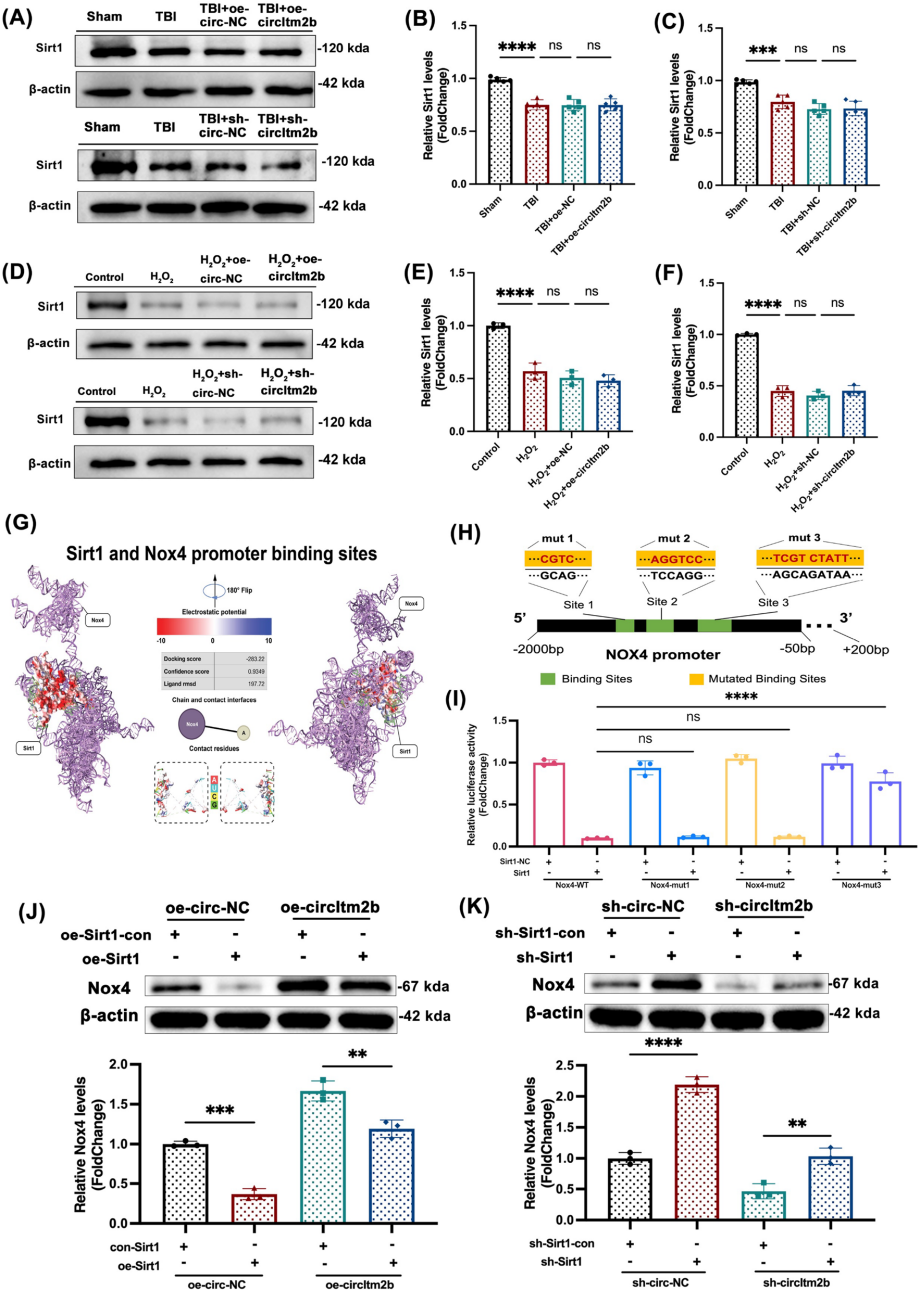
CircItm2b regulates Nox4 expression through binding Sirt1. **A–C** Western blot bands of Sirt1 after circItm2b overexpression or knockdown in mice. The expressions of Sirt1 are detected by western blotting after circItm2b overexpression in mice. n = 5 repetition, TBI vs. Sham group, **** p < 0.0001, TBI + oe-circ-NC vs. TBI, ns not statistically significant, TBI + oe-circItm2b vs. TBI + oe-circ-NC, ns not statistically significant, one-way ANOVA followed by Tukey’s multiple comparisons test**.** The expressions of Sirt1 are detected by western blotting after circItm2b knockdown in mice. n = 5 repetition, TBI vs. Sham group, *** p < 0.001, TBI + sh-circ-NC vs. TBI, ns not statistically significant, TBI + sh-circItm2b vs. TBI + sh-circ-NC, ns not statistically significant, one-way ANOVA followed by Tukey’s multiple comparisons test.** D–F** Western blot bands of Sirt1 after circItm2b overexpression or knockdown in cells. The expressions of Sirt1 are detected by western blotting after circItm2b overexpression in cells. n = 3 repetition, H_2_O_2_ (600 μmol/L) treated HT22 cells for 6 h. H_2_O_2_ vs. Control group, **** p < 0.0001; H_2_O_2_ + oe-circ-NC vs H_2_O_2_, ns not statistically significant; H_2_O_2_ + oe-circItm2b vs. H_2_O_2_ + oe-circ-NC, ns not statistically significant, one-way ANOVA followed by Tukey’s multiple comparisons test**.** The expressions of Sirt1 are detected by western blotting after circItm2b knockdown in cells. n = 3 repetition, H_2_O_2_ (600 μmol/L) treated HT22 cells for 6 h. H_2_O_2_ vs. Control group, **** p < 0.0001; H_2_O_2_ + sh-circ-NC vs H_2_O_2_, ns not statistically significant; H_2_O_2_ + sh-circItm2b vs. H_2_O_2_ + sh-circ-NC, ns not statistically significant, one-way ANOVA followed by Tukey’s multiple comparisons test. **G** The three-dimension docking structure diagram shows the Sirt1 could bind the Nox4 promoter, the docking score: −283.22, the confidence score: 0.9349, the ligand rmsd: 197.72. **H** Schematic illustration of the Nox4 promoter, the green block shows the binding sites and the base sequence, and the yellow block shows the mutated binding sites. **I** Luciferase assay shows the relative luciferase activity in 293 T cells after co-transfection of Nox4-WT/Nox4-mut1/Nox4-mut2/Nox4-mut3 and Sirt1/Sirt1-NC, n = 3 repetition, Nox4-WT + Sirt1 vs. Nox4-mut1 + Sirt1, ns not statistically significant; Nox4-WT + Sirt1 vs. Nox4-mut2 + Sirt1, ns not statistically significant; Nox4-WT + Sirt1 vs. Nox4-mut3 + Sirt1, **** p < 0.0001. One-way ANOVA followed by Tukey’s multiple comparisons test. **J **The expression of Nox4 after co-transfecting the oe-circ-NC + oe-Sirt1-con or oe-circ-NC + oe-Sirt1 or oe-circItm2b + oe-Sirt1-con or oe-circItm2b + oe-Sirt1. n = 3 repetition, oe-circ-NC + oe-Sirt1 vs. oe-circ-NC + oe-Sirt1-con, *** p < 0.001; oe-circItm2b + oe-Sirt1 vs. oe-circItm2b + oe-Sirt1-con, ** p < 0.01. **K** The expression of Nox4 after co-transfecting the sh-circ-NC + sh-Sirt1-con or sh-circ-NC + sh-Sirt1 or sh-circItm2b + sh-Sirt1-con or sh-circItm2b + sh-Sirt1. n = 3 repetition, sh-circ-NC + sh-Sirt1 vs. sh-circ-NC + sh-Sirt1-con, **** p < 0.0001; sh-circItm2b + sh-Sirt1 vs. sh-circItm2b + sh-Sirt1-con, ** p < 0.01. One-way ANOVA followed by Tukey’s multiple comparisons test. All data were represented as mean ± SD.

In addition, Table 2 contains a formatting error. Specifically, the “SCORE” column was mistakenly omitted, which caused the subsequent columns to shift and misalign with their respective headers. This correction is purely technical and does not affect any of the results or conclusions of the study. The incorrect and correct Table 2 is given below.

Incorrect Table 2

**Table 2:** Homology identity of circItm2b between mice and human

Query	STRAT	END	IDENTITY	circRNA	STRAND	START	END
mmu_circ_0005429	151	128	89.20%	hsa_circ_0006620	+	6125	6317
mmu_circ_0005429	146	461	87.70%	hsa_circ_0006620	+	1246	3754
mmu_circ_0005429	26	692	93.60%	hsa_circ_0027605	−	2729	2769
mmu_circ_0005429	26	692	93.60%	hsa_circ_0027608	−	764	804
mmu_circ_0005429	22	691	95.90%	hsa_circ_0012781	+	136709	136,741
mmu_circ_0005429	21	667	65.30%	hsa_circ_0035325	−	67168	67191

Correct Table 2

**Table 2:** Homology identity of circItm2b between mice and human

Query	SCORE	START	END	IDENTITY	circRNA	STRAND	START	END
mmu_circ_0005429	151	128	320	89.20%	hsa_circ_0006620	+	6125	6317
mmu_circ_0005429	146	461	777	87.70%	hsa_circ_0006620	+	1246	3754
mmu_circ_0005429	26	692	724	93.60%	hsa_circ_0027605	−	2729	2769
mmu_circ_0005429	26	692	724	93.60%	hsa_circ_0027608	−	764	804
mmu_circ_0005429	22	691	717	95.90%	hsa_circ_0012781	+	136709	136,741
mmu_circ_0005429	21	667	701	65.30%	hsa_circ_0035325	−	67168	67191
