# Supplementary figures and images for: CircRNA Itm2b induces oxidative stress via the interaction with Sirt1-Nox4 to aggravate sleep disturbances after traumatic brain injury
Source: Cell Biosci. 2025 Feb 17;15:21. doi: 10.1186/s13578-025-01353-6 (PMC11834694; doi:10.1186/s13578-025-01353-6)

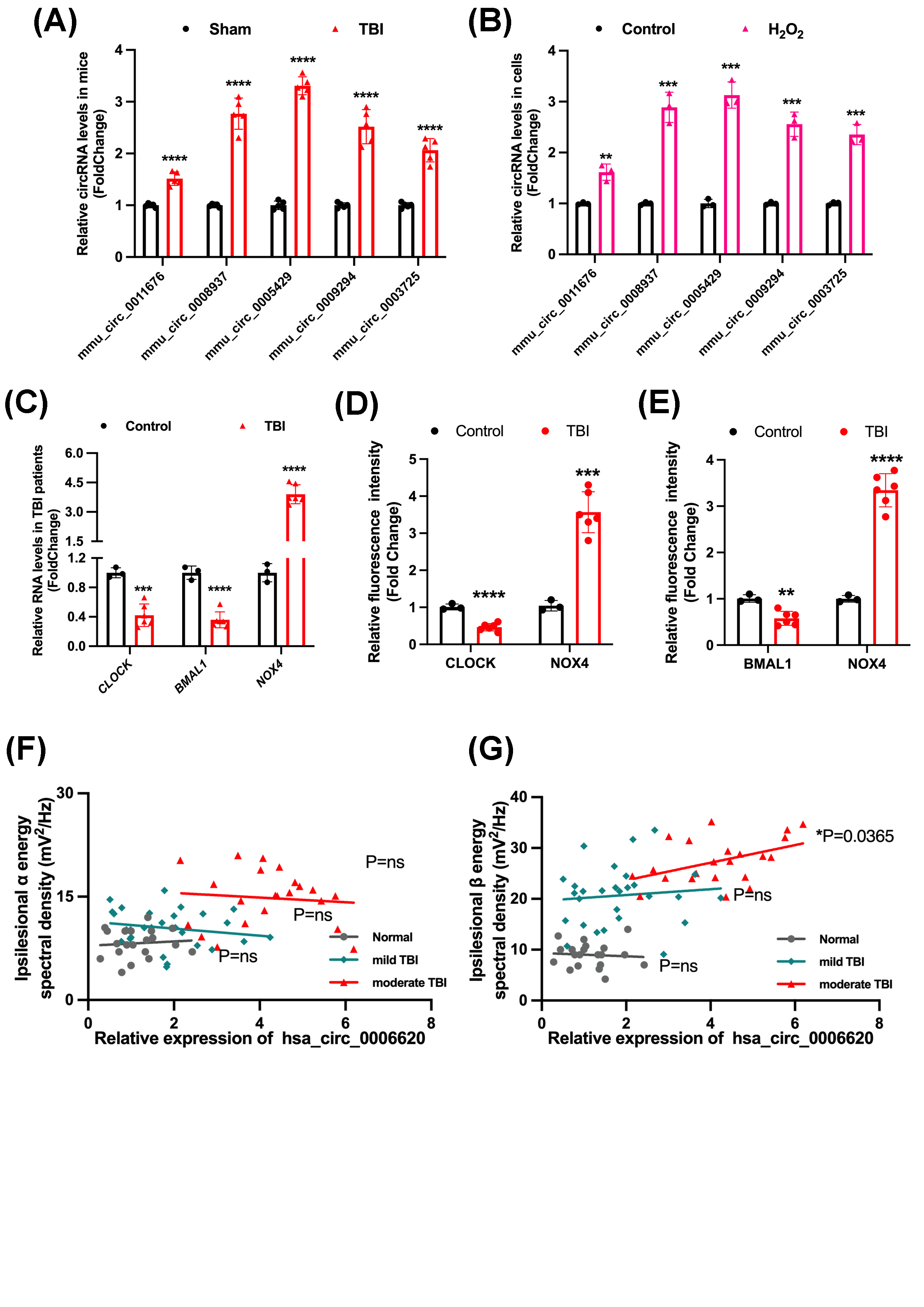

Supplement: Supplementary file 1 — Additional file 1: Figure S1. Relative circRNAs expression in TBI mice and H2O2-treated HT22 cells and the alternation of mRNA or fluorescence intensity and EEG α/β energy density in TBI patients. (A) Relative top 5 up-regulated circRNAs expression levels detected by qRT-PCR in TBI mice brain. n = 5. TBI vs. Sham, **** p < 0.0001. (B) Relative top 5 up-regulated circRNAs expression levels detected by qRT-PCR in HT22 cells treated by H2O2. n = 3. H2O2 vs. Control, ** p < 0.01, *** p < 0.001. (C) Relative CLOCK, BMAL1 and NOX4 mRNA expression levels detected by qRT-PCR in TBI patients brain specimen. TBI patients n = 6, controls n = 3. TBI vs. Control, *** p < 0.001, **** p < 0.0001. (D-E) Relative fluorescence intensity in double immunofluorescence show the intensity of NOX4, CLOCK or BMAL1 in patients with acute TBI. TBI patients n = 6, controls n = 3. TBI vs. Control, ** p < 0.01, *** p < 0.001, **** p < 0.0001. One-way ANOVA followed by Tukey’s multiple comparisons test. (F-G) The correlation analysis between alpha, beta energy density in sleep wake cycle and expression of hsa_circ_0006620 in the serum of TBI patients are shown. Grey points and lines represent the normal group; green points and lines represent the mild TBI group; red points and lines represent the moderate TBI group. All data were represented as mean ± SD. [file 13578_2025_1353_MOESM1_ESM.tif]

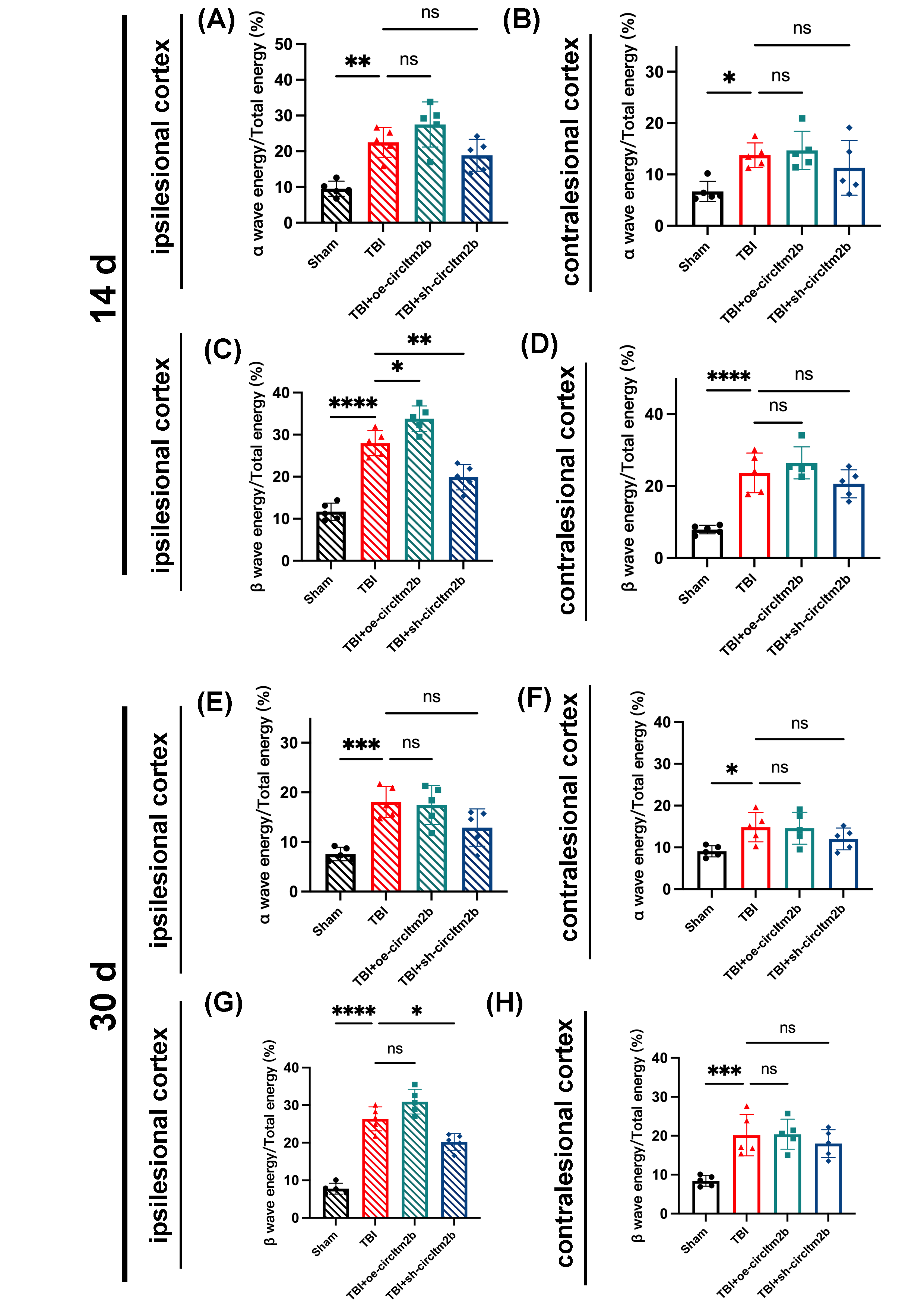

Supplement: Supplementary file 2 — Additional file 2: Figure S2. Relative EEG α/β energy density changes in TBI mice on 14d and 30d. (A-B) Relative alpha, energy density in the ipsilesional and contralesional cortex at 14d after TBI. n = 5 mice per group. (C-D) Relative beta energy density in the ipsilesional and contralesional cortex at 14d after TBI. n = 5 mice per group. (E-F) Relative alpha energy density in the ipsilesional and contralesional cortex at 30d after TBI. n = 5 mice per group. (G-H) Relative beta energy density in the ipsilesional and contralesional cortex at 30d after TBI. n = 5 mice per group. One-way ANOVA followed by Tukey’s multiple comparisons test. All data were represented as mean ± SD [file 13578_2025_1353_MOESM2_ESM.tif]

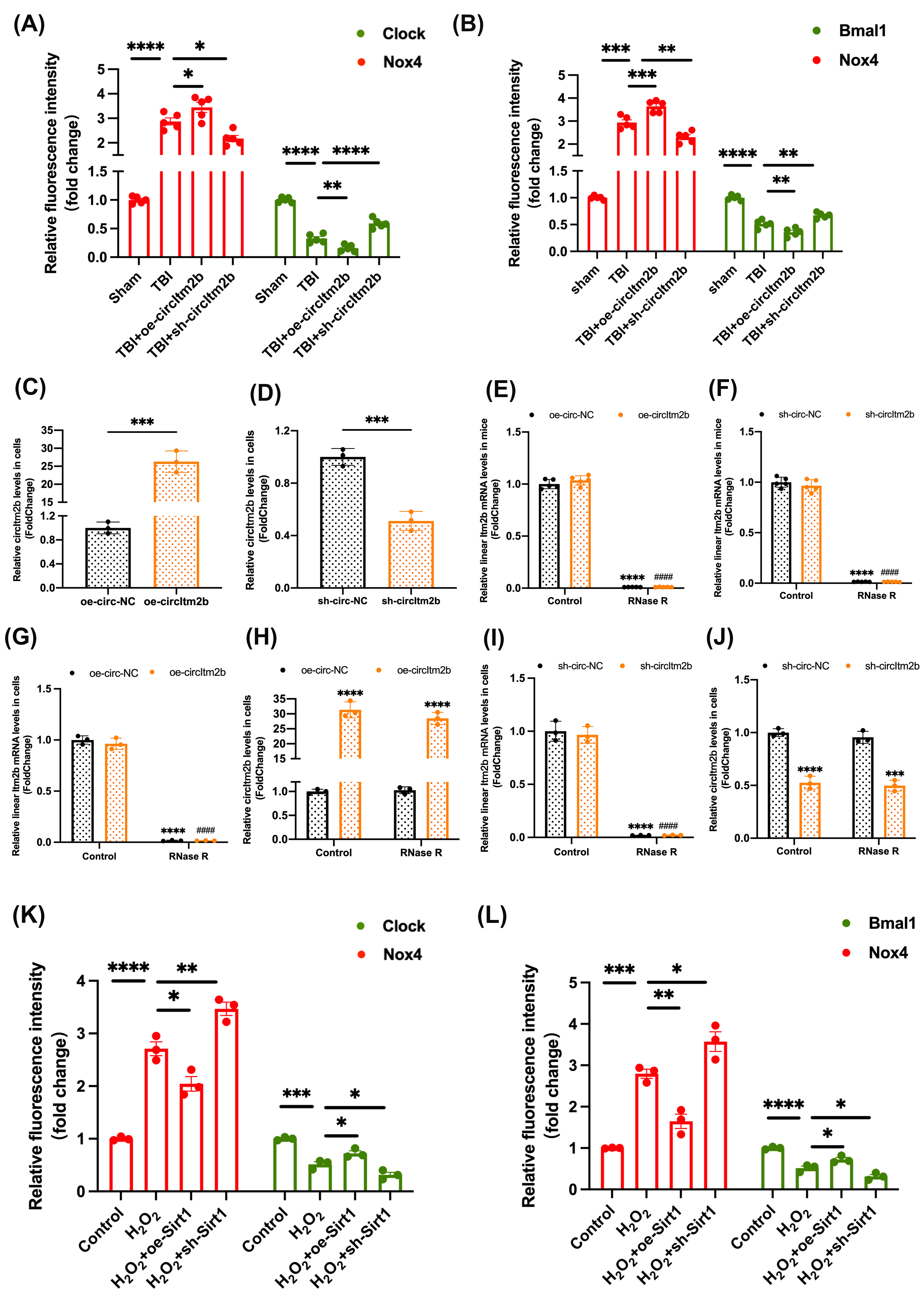

Supplement: Supplementary file 3 — Additional file 3: Figure S3. The relative fluorescence intensity alternations and identification of circular structure of circItm2b. (A) Relative fluorescence intensity in double immunofluorescence shows the intensity of Nox4 and Clock in TBI mice brain. n = 5, Nox4: TBI vs. Sham, **** p < 0.0001, TBI + oe-circItm2b vs. TBI, * p < 0.01, TBI + sh-circItm2b vs. TBI, * p < 0.01. Clock: TBI vs. Sham, **** p < 0.0001, TBI + oe-circItm2b vs. TBI, ** p < 0.01, TBI + sh-circItm2b vs. TBI, **** p < 0.0001. (B) Relative fluorescence intensity in double immunofluorescence shows the intensity of Nox4 and Bmal1 in TBI mice brain. n = 5, Nox4: TBI vs. Sham, *** p < 0.001, TBI + oe-circItm2b vs. TBI, *** p < 0.001, TBI + sh-circItm2b vs. TBI, ** p < 0.01. Bmal1: TBI vs. Sham, **** p < 0.0001, TBI + oe-circItm2b vs. TBI, ** p < 0.01, TBI + sh-circItm2b vs. TBI, ** p < 0.01. (C) Relative circItm2b expression level detected by qRT-PCR in HT22 cells after the transduction of overexpression lentivirus for 7 days. n = 3 replications, *** p < 0.001, two-tailed t-test. (D) Relative circItm2b expression level detected by qRT-PCR in HT22 cells after the transduction of knockdown lentivirus for 7 days. n = 3 replications, *** p < 0.001, two-tailed t-test. (E) Relative linear Itm2b mRNA expression detected by qRT-PCR in mice after oe-circItm2b lentivirus transduction by Rnase R. n = 5 replications. Rnase R + oe-circ-NC vs. Control + oe-circ-NC, **** p < 0.0001, Rnase R + oe-circItm2b vs. Control + oe-circItm2b, #### p < 0.0001. Two-way ANOVA followed by Tukey’s multiple comparisons test. (F) Relative linear Itm2b mRNA expression detected by qRT-PCR in mice after sh-circItm2b lentivirus transduction by Rnase R. n = 5 replications. Rnase R + sh-circ-NC vs. Control + sh-circ-NC, **** p < 0.0001, Rnase R + sh-circItm2b vs. Control + sh-circItm2b, #### p < 0.0001. Two-way ANOVA followed by Tukey’s multiple comparisons test. (G) Relative linear Itm2b mRNA expression detected by qRT-PCR in HT22 cel [file 13578_2025_1353_MOESM3_ESM.tif]
