# Supplementary material for: CircRNA Itm2b induces oxidative stress via the interaction with Sirt1-Nox4 to aggravate sleep disturbances after traumatic brain injury
Source: Cell Biosci. 2025 Feb 17;15:21. doi: 10.1186/s13578-025-01353-6 (PMC11834694; doi:10.1186/s13578-025-01353-6)
Supplement: Supplementary file 4 — Additional file 4: Table S1. Clinical data from control and TBI cases. [file 13578_2025_1353_MOESM4_ESM.docx]

**Supplementary Table 1.** Clinical data from control and TBI cases.

| NO. | Group | Gender | Age | Injury Site | Injury Duration | GCS |
| --- | --- | --- | --- | --- | --- | --- |
| 1 | Control | F | 33 | / | / | 15 |
| 2 | Control | F | 48 | / | / | 15 |
| 3 | Control | M | 31 | / | / | 15 |
| 4 | Control | F | 49 | / | / | 15 |
| 5 | Control | M | 55 | / | / | 15 |
| 6 | Control | F | 37 | / | / | 15 |
| 7 | Control | F | 39 | / | / | 15 |
| 8 | Control | M | 42 | / | / | 15 |
| 9 | Control | M | 44 | / | / | 15 |
| 10 | Control | M | 46 | / | / | 15 |
| 11 | Control | F | 27 | / | / | 15 |
| 12 | Control | M | 20 | / | / | 15 |
| 13 | Control | F | 39 | / | / | 15 |
| 14 | Control | F | 24 | / | / | 15 |
| 15 | Control | M | 50 | / | / | 15 |
| 16 | Control | M | 32 | / | / | 15 |
| 17 | Control | F | 37 | / | / | 15 |
| 18 | Control | M | 35 | / | / | 15 |
| 19 | Control | F | 40 | / | / | 15 |
| 20 | Control | M | 46 | / | / | 15 |
| 21 | TBI | F | 59 | Frontal lobe | 8h | 14 |
| 22 | TBI | M | 22 | Frontotemporal lobe | 1d | 8 |
| 23 | TBI | F | 61 | Frontal Parietal lobe | 10h | 14 |
| 24 | TBI | M | 51 | Temporal lobe | 2h | 11 |
| 25 | TBI | M | 65 | Frontal lobe | 7h | 12 |
| 26 | TBI | M | 18 | Frontal Parietal lobe | 6h | 14 |
| 27 | TBI | F | 55 | Parietal lobe | 1d | 15 |
| 28 | TBI | M | 56 | Temporal lobe | 4h | 14 |
| 29 | TBI | M | 40 | Frontotemporal lobe | 5h | 13 |
| 30 | TBI | M | 26 | Temporal lobe | 1d | 10 |
| 31 | TBI | M | 52 | Frontal lobe | 3h | 14 |
| 32 | TBI | M | 51 | Frontotemporal lobe | 10h | 9 |
| 33 | TBI | F | 65 | Frontal Parietal lobe | 3d | 15 |
| 34 | TBI | F | 53 | Frontal lobe | 9h | 14 |
| 35 | TBI | M | 56 | Temporal lobe | 18h | 15 |
| 36 | TBI | F | 37 | Parietal lobe | 6h | 12 |
| 37 | TBI | M | 18 | Frontotemporal lobe | 2d | 14 |
| 38 | TBI | M | 62 | Frontal lobe | 5h | 11 |
| 39 | TBI | F | 18 | Frontal Parietal lobe | 6h | 9 |
| 40 | TBI | M | 60 | Frontal lobe | 2h | 14 |
| 41 | TBI | M | 58 | Frontal lobe | 1d | 15 |
| 42 | TBI | M | 47 | Frontal Parietal lobe | 4h | 14 |
| 43 | TBI | F | 35 | Temporal lobe | 8h | 15 |
| 44 | TBI | M | 55 | Frontal lobe | 3h | 9 |
| 45 | TBI | F | 53 | Frontotemporal lobe | 9h | 15 |
| 46 | TBI | F | 64 | Parietal lobe | 1d | 14 |
| 47 | TBI | M | 56 | Temporal lobe | 1h | 15 |
| 48 | TBI | M | 49 | Frontotemporal lobe | 6h | 12 |
| 49 | TBI | F | 42 | Frontal lobe | 12h | 11 |
| 50 | TBI | F | 54 | Temporal lobe | 20h | 8 |
| 51 | TBI | M | 51 | Frontal lobe | 3d | 11 |
| 52 | TBI | F | 19 | Frontal Parietal lobe | 16h | 15 |
| 53 | TBI | M | 34 | Frontal lobe | 4h | 12 |
| 54 | TBI | M | 61 | Temporal lobe | 2h | 14 |
| 55 | TBI | F | 48 | Frontal Parietal lobe | 2d | 12 |
| 56 | TBI | M | 35 | Frontotemporal lobe | 10h | 14 |
| 57 | TBI | F | 57 | Frontal lobe | 5h | 15 |
| 58 | TBI | M | 19 | Temporal lobe | 3h | 9 |
| 59 | TBI | F | 53 | Frontal lobe | 1d | 10 |
| 60 | TBI | M | 45 | Frontotemporal lobe | 13h | 14 |
| 61 | TBI | M | 58 | Parietal lobe | 6h | 9 |
| 62 | TBI | F | 63 | Temporal lobe | 8h | 13 |
| 63 | TBI | M | 18 | Frontal Parietal lobe | 10h | 14 |
| 64 | TBI | M | 65 | Frontal lobe | 1d | 13 |
| 65 | TBI | F | 29 | Frontotemporal lobe | 6h | 9 |
| 66 | TBI | M | 55 | Frontal lobe | 16h | 12 |
| 67 | TBI | M | 21 | Temporal lobe | 3d | 11 |
| 68 | TBI | F | 26 | Frontal Parietal lobe | 10h | 12 |
| 69 | TBI | F | 57 | Frontal lobe | 8h | 15 |
| 70 | TBI | M | 19 | Frontotemporal lobe | 4h | 13 |
