# Supplementary material for: CircRNA Itm2b induces oxidative stress via the interaction with Sirt1-Nox4 to aggravate sleep disturbances after traumatic brain injury
Source: Cell Biosci. 2025 Feb 17;15:21. doi: 10.1186/s13578-025-01353-6 (PMC11834694; doi:10.1186/s13578-025-01353-6)
Supplement: Supplementary file 5 — Additional file 5: Table S2. The binding score between circItm2b and SIRT1. [file 13578_2025_1353_MOESM5_ESM.docx]

|  | Protein region | RNA region | Interaction Propensity | Discriminative Power | Normalized Score |
| --- | --- | --- | --- | --- | --- |
| 1 | 637-688 | 56-107 | 16.17 | 45 | 5.52 |
| 2 | 637-688 | 51-102 | 12.89 | 35 | 4.46 |
| 3 | 637-688 | 31-82 | 11.87 | 33 | 4.13 |
| 4 | 226-277 | 56-107 | 11.6 | 33 | 4.04 |
| 5 | 637-688 | 26-77 | 10.82 | 32 | 3.79 |
| 6 | 112-163 | 56-107 | 10.57 | 32 | 3.71 |
| 7 | 76-127 | 56-107 | 10.46 | 32 | 3.67 |
| 8 | 462-513 | 56-107 | 10.33 | 32 | 3.63 |
| 9 | 62-113 | 56-107 | 9.79 | 28 | 3.46 |
| 10 | 226-277 | 51-102 | 9.61 | 28 | 3.4 |
| 11 | 237-288 | 56-107 | 9.45 | 28 | 3.35 |
| 12 | 637-688 | 81-132 | 9.16 | 28 | 3.25 |
| 13 | 501-552 | 56-107 | 9.04 | 28 | 3.21 |
| 14 | 362-413 | 56-107 | 8.79 | 26 | 3.13 |
| 15 | 76-127 | 51-102 | 8.76 | 26 | 3.12 |
| 16 | 412-463 | 56-107 | 8.57 | 26 | 3.06 |
| 17 | 326-377 | 56-107 | 8.56 | 26 | 3.06 |
| 18 | 112-163 | 51-102 | 8.48 | 26 | 3.03 |
| 19 | 462-513 | 51-102 | 8.45 | 26 | 3.02 |
| 20 | 451-502 | 56-107 | 8.34 | 26 | 2.99 |

**Supplementary Table 2.** The binding score between circItm2b and Sirt1.
